# Napthrene Compounds from Mycelial Fermentation Products of *Marasmius berteroi*

**DOI:** 10.3390/molecules25173898

**Published:** 2020-08-26

**Authors:** Ning Ning Yang, Qing Yun Ma, Fan Dong Kong, Qing Yi Xie, Hao Fu Dai, Li Man Zhou, Zhi Fang Yu, You Xing Zhao

**Affiliations:** 1 Hainan Key Laboratory for Research and Development of Natural Product from Li Folk Medicine, Institute of Tropical Bioscience and Biotechnology, Chinese Academy of Tropical Agricultural Sciences, Haikou 571101, China; yangningning891216@163.com (N.N.Y.); maqingyun@itbb.org.cn (Q.Y.M.); kongfandong@itbb.org.cn (F.D.K.); xieqingyi@itbb.org.cn (Q.Y.X.); daihaofu@itbb.org.cn (H.F.D.); zhouliman88@163.com (L.M.Z.); 2 College of Food and Bioengineering, Bengbu University, Bengbu 233030, China; 3 Hainan Institute for Tropical Agricultural Resources, Chinese Academy of Tropical Agricultural Sciences, Haikou 571101, China; 4 College of Food Science and Technology, Nanjing Agricultural University, Nanjing 210095, China

**Keywords:** *Marasmius berteroi*, dipolynaphthalenes, binapthrene, AChE inhibitory activity, cytotoxic activity

## Abstract

The metabolites of the genus *Marasmius* are diverse, showing good research prospects for finding new bioactive molecules. In order to explore the active metabolites of the fungi *Marasmius berteroi*, the deep chemical investigation on the bioactive compounds from its cultures was undertaken, which led to the isolation of three new naphthalene compounds dipolynaphthalenes A–B (**1**,**2**) and naphthone C (**3**), as well as 12 known compounds (**4**–**15**). Compounds **1**, **2**, and **4** are dimeric naphthalene compounds. Their structures were elucidated by MS, 1D and 2D NMR spectroscopic data, as well as ECD calculations. Compounds **2**–**4** and **7** exhibited acetylcholinesterase (AChE) inhibitory activities at the concentration of 50 μg/mL with inhibition ratios of 42.74%, 44.63%, 39.50% and 51.49%, respectively. Compounds **5** and **7**,**8** showed weak inhibitory activities towards two tumor cell lines, with IC_50_ of 0.10, 0.076 and 0.058 mM (K562) and 0.13, 0.18, and 0.15 mM (SGC-7901), respectively.

## 1. Introduction

Natural products are an important source of innovative chemical drugs [1]. Finding natural active ingredients for nerve protection and tumor suppression are also hot topics in the current research [2]. The genus *Marasmius* is a common basidiomycete in tropical and subtropical areas, which belongs to the family *Marasmiaceae* [3]. A few common species of *Marasmius* have been studied for searching of bioactive metabolites, and some active compounds such as terpenoids [4,5,6,7,8], steroids [9], cyclic peptides [10], isocoumarins [11] and piperidones [12] have been isolated. Many of these compounds showed antibacterial, cytotoxic and antihypertensive activities [9,10,11,12]. *Marasmius berteroi* is a small orange mushroom belonging to the genus *Marasmius*, which is widely distributed in the southeast of mainland China, mainly in Hainan, Guangdong, and Taiwan provinces. So far, few researches on chemical constituents of this fungus were reported [13]. To seek for new active small molecules from *Marasmius berteroi*, the deep chemical investigation on its cultures was thus undertaken, which led to the isolation of three new naphthalene ring compounds dipolynaphthalenes A–B (**1**,**2**) and naphthone C (**3**) along with twelve known analogues (**4**–**15**). The isolation process and structural elucidation of three new compounds, as well as their AChE inhibitory and cytotoxic activity, are described in this paper.

## 2. Results and Discussion

**Figure 1:**
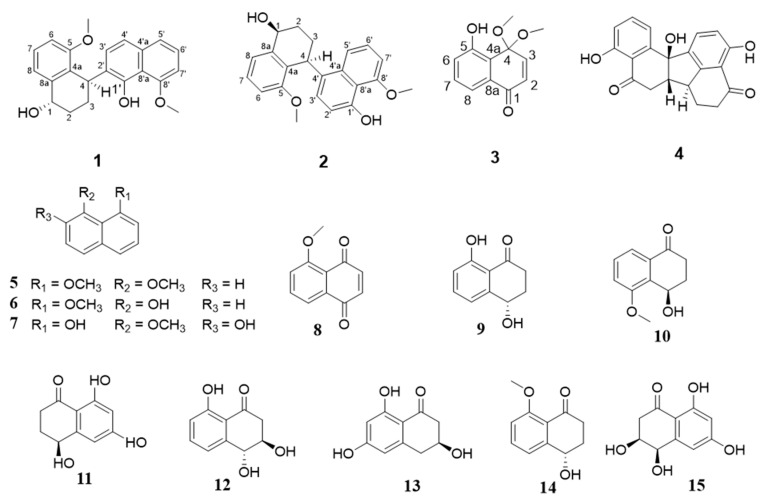
The structures of compounds **1*–*15**.

**Figure 2:**
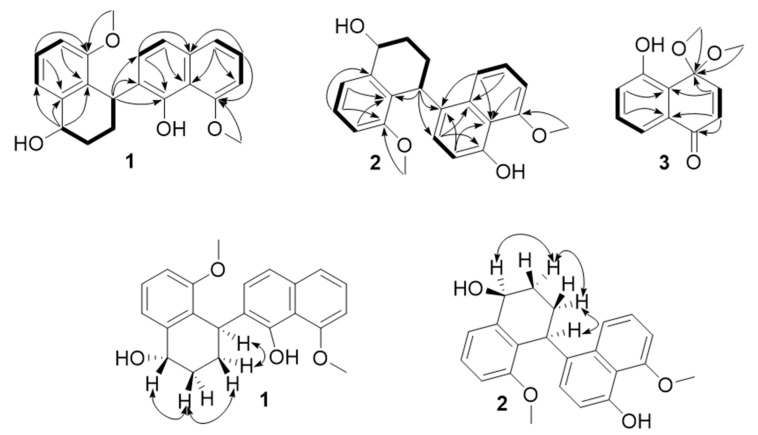
Key ^1^H-^1^H COSY (▬), HMBC (H→C), and ROESY (↔) correlations of **1**–**3**.

Dipolynaphthalene A (compound **1**) was isolated as pale-yellow oil. Its molecular formula was assigned to be C_22_H_22_O_4_ with twelve degrees of unsaturation according to its positive HR-ESI-MS (*m/z* 373.1412 [M + Na]^+^, calcd. 373.1410 for C_22_H_22_O_4_Na) and NMR spectroscopic data (Table 1). The IR (Supplementary Materials) spectrum revealed the presence of hydroxyl (3305 cm^−1^) and benzene ring (1581cm^−1^) absorptions. The ^13^C-NMR and DEPT spectroscopic data (Table 1) showed 22 carbon resonances, including two methoxyls, two sp^3^ methylenes, ten methines (eight olefinic and one oxygenated), and eight quaternary carbons (three oxygenated). The ^1^H-NMR (Table 1) showed the presence of signals for two singlet methoxys (*δ* 4.06 (3H, s, H-5), 4.08 (3H, s, H-8′)). According to a comparison of the corresponding NMR data, compound **1** was similar to nodulisporin B [14], a dimer of naphthalene ring compound, except for the two naphthalene rings have different binding sites. Compound **1** was 4,2′-binaphthalene, while nodulisporin B was 2,2′-binaphthalene, which was evidenced by the key HMBC correlations from H-3 (δ 2.12 m, 2.21 m) to quaternary carbon C-2′ (*δ*_C_ 128.1), H-4 (δ 4.74 m) to quaternary carbon C-1′ (*δ*_C_ 150.4). Other correlations in the HMBC (C-5/H-6/H-7/OCH_3_, C-1/H-2, H-1/C-8a/C-2, C-1′/H-4, C-8′/H-7′/H-6′) and ^1^H–^1^H COSY spectra further supported the atom connectivity in compound **1** (Figure 1 and Figure 2). The relative configuration of compound **1** was determined by ROESY cross-peaks H-4[*δ*_H_ 4.74 (1H, m)]/H-3*α*[*δ*_H_ 2.12 (1H, m)]/H-2*α*[*δ*_H_ 1.75 (1H, m)]/H-3′[*δ*_H_ 7.23 (1H, d, *J* = 8.1)], H-3*β*[*δ*_H_ 2.21 (1H, m)]/H-2*β*[*δ*_H_ 1.95 (1H, m)]/H-1 [*δ*_H_ 4.85 (1H, dd, *J* = 5.4, 8.8 Hz)]. Thus, compound **1** was assigned as shown in Figure 1 and it was named dipolynaphthalene A.

Dipolynaphthalene B (compound **2**) was purified as pale yellow oil, and possessed the molecular formula C_22_H_22_O_4_ based on HR-ESI-MS (*m*/*z* 373.1410 [M + Na]^+^, calcd for C_22_H_22_O_4_Na, 373.1410) with twelve degrees of unsaturation. The IR spectrum showed the presence of hydroxyl (3399 cm^−1^) and benzene ring (1583 cm^−1^) absorptions. The ^13^C-NMR spectrum of **2** displayed 22 carbon resonances extremely similar to those of compound **1**, suggesting that both of them were dimeric naphthalene rings. The only difference was that compound **2** was 4,4′-binaphthalene instead of 4,2′-binaphthalene in **1**, which was further supported by the HMBC correlations (Figure 2) from H-4 [*δ* 5.03 d (5.4)] to C-3′ (*δ*_C_ 126.8) and C-4′a (*δ*_C_ 131.6), H-3 (*δ* 1.85 m) to C-4′ (*δ*_C_ 128.3). Other correlations in the HMBC and ^1^H–^1^H COSY spectra (Figure 2) further supported the atom connectivity in compound **2**. The configurations of 1-*α* OH and 4-*β* H in **2** were deduced from its ROESY cross-peaks (Figure 2) H-1*α/*H-2*α* [*δ*_H_ 1.81 (1H, m)]/H-3*α*[*δ*_H_ 2.37 (1H, m)]/H-4 [*δ*_H_ 5.03 (1H, d, *J* = 5.4 Hz)]. Thus, compound **2** was assigned as shown in Figure 1, and named dipolynaphthalene B.

**Figure 3:**
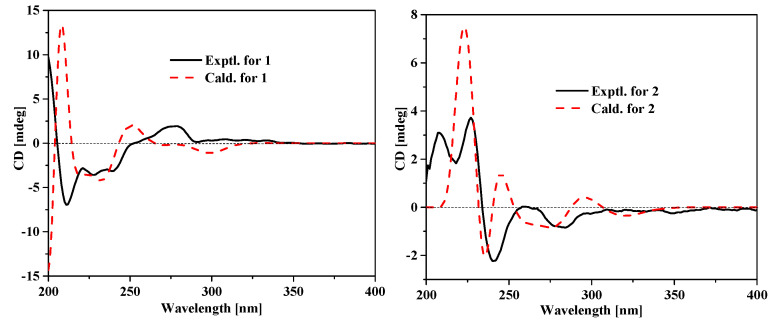
Experimental and calculated ECD spectra for compounds **1** and **2**.

In order to determine the absolute configurations of **1** and **2**, the ECD spectra were calculated by the TDDFT method at the apfd/6-311+g (2d, p) level. The calculated ECD spectra of **1** and **2** are generally consistent with their measured spectra (Figure 3), implying the (1*S*,4*R*)- configuration for **1** and the (1*S*,4*S*)- configuration for **2** (Figure 3). However, much more work was needed to confirm the absolute configurations of **1** and **2**, for there are also some differences present between the calculated and measured ECD spectra.

Naphthone C (**3**) was isolated as pale yellow oil, which molecular formula was assigned to be C_12_H_12_O_4_ with seven degrees of unsaturation according to its positive HR-ESI-MS (*m*/*z* 243.0622 [M + Na]^+^, calcd. 243.0628 for C_12_H_12_O_4_Na) and NMR spectroscopic data (Table 2). The IR spectrum revealed the presence of hydroxyl (3323 cm^−1^), carbonyl (1714 cm^−1^) and double bond (1677cm^−1^) absorptions. The ^13^C-NMR and DEPT spectroscopic data (Table 2) showed 12 carbon resonances, including two methoxyls, five methines, and five quaternary carbons (two oxygenated and one carbonyl carbon), which was inferred that the basic skeleton of compound **3** was naphthone. The ^1^H-NMR (Table 2) showed the presence of signals for singlet methoxy (*δ* 3.35 (3H, s, H-4)). According to a comparison of the corresponding NMR data, compound **3** was similar to 4,4,5-trimethoxy-1(4*H*)-naphthone [15], a derivative of naphthalene ring compound, except for the hydroxyl (C-5 *δ*_C_157.9) in **3** instead of methoxy in 4,4,5-trimethoxy-1(4*H*)-naphthone, which was further supported by the HMBC correlations (Figure 2) from H-6 [*δ*_H_ 6.94 dd (8.3,1.06)] to C-5 (*δ*_C_ 157.9) and H-7 [*δ*_H_ 7.27 t (7.5)] to C-5. Other correlations in the HMBC further supported the atom connectivity in compound. Thus, compound **3** was assigned as shown in Figure 1, and named naphthone C.

The twelve known compounds (**4**–**15**) was determined to be daldinone C (**4**) [16], 1,8-dimethoxynaphthalene (**5**) [17], 8-methoxy-1-naphthol (**6**) [18], 8-methoxynaphthalen e-1,7 -diol (**7**) [19], 5-methoxy-1,4-naphthoquinone (**8**) [20], isosclerone (**9**) [21], 5-*O*-methylsclerone (**10**) [22] 4,6,8-trihydroxy-3,4-dihydronaphthalen-1(2*H*)-one(6-hydroxy-isosclerone) (**11**) [23], *trans*-3,4-dihydro-3,4,8-trihydroxynaphthalene-1-(2*H*)-one(**12**) [24], scytalone (**13**) [25], xylarenone (**14**) [26] and *cis*-4-hydroxyscytalone (**15**) [27] by comparison of its spectroscopic data with those of literature.

The acetylcholinesterase inhibitory activity of compounds **1**–**15** were tested by previous method as described in the literature [28]. The results showed that compounds **2**–**4** and compound **7** exhibited anti-acetylcholinesterase activities at a concentration of 50 μg/mL with inhibition ratios of 42.74%, 44.63%, 9.50%, and 51.49%, respectively (Table 3). In the screening test of cytotoxicity [29], compounds **5**, **7**, **8** showed inhibitory activity against two tumor cell lines, with IC_50_ of 0.10, 0.076 and 0.058 mM (K562) and 0.13, 0.18, and 0.15 mM (SGC-7901), respectively (Table 4). Binaphthalenes are dimeric compounds of naphthalene ring, mainly derivatives of DHN and juglone formed by different linking modes [30]. The acetylcholinesterase inhibitory activity evaluation results showed that compounds **2** and **4** exhibited anti-acetylcholinesterase activities, while compound **1** was inactive. Structurally, the active compounds **2** and **4** were naphthalene ring dimers with para-para ligation, while compound **1** was ortho-para ligation dimer, which indicated that the polymeric sites in binaphthalene compounds were very important for their biological activity. In the cytotoxic assay, compound **8** were active, while **9**–**15** were inactive, which may suggest that the 1,4-dione moiety is essential for their cytotoxicity.

## 3. Materials and Methods

### 3.1. General Information

Optical rotations were measured with a Rudolph Autopol III polarimeter (Rudolph Research Analytical, Hackettstown, NJ, USA). Shimadzu UV-2550 spectrometer (Beckman, Brea, CA, USA) was used for scanning UV spectroscopy. IR spectra were obtained on a Tensor 27 spectrometer, as KBr pellets (Thermo, Pittsburgh, PA, USA). NMR spectra were recorded on an AV-500 spectrometer (Bruker, Bremen, Germany) with TMS (Tetramethylsilane) as an internal standard. HR-ESI-MS were performed on an API QSTAR Pulsar mass spectrometer (Billerica, MA, USA). Silica gel (200–300 mesh, Qingdao Marine Chemical Inc., Qingdao, China), RP-18 (40–70 mm, Fuji Silysia Chemical Ltd., Kasugai Aichi, Japan) and Sephadex LH-20 (GE Healthcare, Uppsala, Sweden) were used for column chromatography (CC). Semipreparative HPLC (Agilent 1100, Agilent Technologies Inc., Santa Clara, CA, USA) was performed on an Agilent 1100 liquid chromatograph with a Zorbax SB-C_18_, 9.4 mm × 25 cm, column. Fractions were monitored by TLC and spots were visualized by heating after spraying with 5% H_2_SO_4_ in ethanol.

### 3.2. Fungal Material

The fungus *Marasmius berteroi* was collected in the valley of Yinggeling, Hainan province of China, in November 2015, and identified by Associate Professor Sheng-zhuo Huang, the Institute of Tropical Bioscience and Biotechnology, Chinese Academy of Tropical Agricultural Sciences. The mycelium was isolated from the *Marasmius berteroi* and its strain was maintained on potato dextrose agar (PDA) slant at 4 °C. A voucher specimen (YGL-18) was deposited at the Institute of Tropical Bioscience and Biotechnology, Chinese Academy of Tropical Agricultural Sciences, Haikou, China.

### 3.3. Fermentation, Extraction and Isolation

The fungus was cultured on slants of PDA medium at 28 °C for 5 days. Plugs of agar supporting mycelium growth were cut and transferred aseptically to 200 × 1000 mL Erlenmeyer flasks each containing 300 mL medium. The flask was incubated at room temperature under static conditions for 30 days. The culture broth (60 L) was filtered to give the filtrate and mycelia. The filtrate was evaporated in vacuo to a small volume and then suspended in H_2_O and partitioned successively with EtOAc and n-BuOH. The EtOAc solution was evaporated under reduced pressure to give a crude extract (32.0 g), which was separated into fractions 1–6 on silica gel CC using a gradient eluent of petroleum ether-EtOAc (15:1–1:1, *v*/*v*, each 3 L). Fr. 2 (7.5 g) was subjected to repeated RP-18 CC (eluted with MeOH/H_2_O from 2:8 to 10:0, *v*/*v*, each 500 mL) and silica gel CC (eluted with petroleum ether–EtOAc from 5:1 to 1:1, *v*/*v*, each 600 mL) to afford compounds **1** (7.0 mg), **2** (5.0 mg) and **4** (3.0 mg). Fr. 3 (6.0 g) was applied to octadecyl silane (ODS) gel with gradient elution of MeOH–H_2_O (1:5, 2:3, 3:2, 4:1, 1:0) to yield compounds **3** (4.0 mg), **6** (8.0 mg) and **7** (2.0 mg). Fr. 4 (5.0 g) was purified by HPLC over an ODS column (30–85% MeOH/H_2_O, *v*/*v*) to give compounds **5** (4 mg), **8** (8.0 mg), **9** (2.0 mg) and **11** (2.0 mg). Fr.5 (3.5 g) was purified by HPLC over an ODS column (30–75% MeOH/H_2_O, *v*/*v*) to give compound **10** (4 mg) and **12** (2 mg). The compounds **13** (3 mg), **14** (9.0 mg), **15** (4.0 mg) were purified from Fr.6 (5 g) by HPLC over an ODS column (30–75% MeOH/H_2_O, *v*/*v*).

#### 3.3.1. Dipolynaphthalene A (1)

Pale yellow oil; [*α*]25D − 14 (*c* 0.10, MeOH); IR (KBr) *ν*_max_ 3305, 2947, 2346, 2301, 1735, 1661, 1581, 1459, 1258, 1015 cm^−1^; ^1^H- and ^13^C-NMR data see Table 1; ESI-MS positive *m/z* [M + Na]^+^ 373; HR-ESI-MS *m/z* [M + Na]^+^ 373.1412 (calcd for C_22_H_22_O_4_Na, 373.1410).

#### 3.3.2. Dipolynaphthalene B (2)

Pale yellow oil; [*α*]25D − 8 (*c* 0.10, MeOH); IR (KBr) *ν*_max_ 3399, 2943, 2366, 2344, 1657, 1618, 1583, 1467, 1407, 1258 cm^−1^; ^1^H- and ^13^C-NMR data see Table 1; ESI-MS positive *m/z* [M + Na]^+^ 373; HR-ESI-MS *m/z* [M + Na]^+^ 373.1410 (calcd for C_22_H_22_O_4_Na, 373.1410).

#### 3.3.3. Naphthone C (3)

Pale yellow oil; [*α*]25D − 42.9 (*c* 0.15, MeOH); IR (KBr) *ν*_max_ 3323, 2354, 2319, 1714, 1677, 1577, 1461, 1273 cm^−1^; ^1^H- and ^13^C-NMR data see Table 2; ESI-MS positive *m/z* [M + Na]^+^ 243; HR-ESI-MS *m/z* [M + Na]^+^ 243.0622 (calcd for C_15_H_24_O_5_Na, 243.0628).

### 3.4. Bioassay of AChE Inhibitory Activity and Cytotoxic Activity

AChE inhibitory activity of these compounds was assayed by the spectrophotometric method developed by Ellman [28]. Acetylthiocholine iodide (Sigma, St. Louis, MO, USA) was used as substrate in the assay. Na_2_HPO_4_ (94.7 mL, 0.1 M) and NaH_2_PO_4_ (5.3 mL, 0.1 M) were mixed to get phosphate buffer (PB, pH 8.0). Compounds were dissolved in DMSO (2% in PB). The reaction mixture contained PB (110 μL), test compound solution (10 μL, 2000 μM) and acetyl cholinesterase solution (40 μL, 0.1 U/mL), which were mixed and incubated for 20 min (30 °C). The reaction was initiated by the addition of DTNB (5,5-dithiobis-2-nitrobenzoic acid, 20 μL, 6.25 mM) and acetylthiocholine iodide (20 μL, 6.25 mM). The hydrolysis of acetylthiocholine was monitored at 405 nm every 30 s. Tacrine (Sigma-Aldrich 99%) was used as positive control (final concentration 0.33 μM), while 2% DMSO in PB was set as negative control (NC). All reactions were performed in triplicate. The percentage inhibition was calculated as follows: % age inhibition = (*E* − *S*)/*E* × 100 (*E* is activity of the enzyme without test compound and *S* is the activity of enzyme with test compound).

The cytotoxicities of compounds **1**–**15** (purity of tested compounds over 95%) were assessed using the MTT [3-4,5-dimethylthiazol-2-yl)-2,5-diphenyltetrazolium bromide] assay against human cell lines K562, BEL7702, andSGC7901 according to the method described in previous literature [29] Taxol (Kyowa Hakko Kogyo Co., Tokyo, Japan; purity 99%) was used as a positive control.

## 4. Conclusions

The secondary metabolites of fungi were various, which were good sources of active substances. The special compounds isolated from *Marasmius berteroi* also showed the diversity of the genus *Marasmius*. In the present study, three new naphthalene compounds dipolynaphthalenes A–B (**1**–**2**) and naphthone C (**3**), as well as 12 known compounds were isolated from the mycelial fermentation products of *Marasmius berteroi*. The bioactivity evaluation assays showed that dipolynaphthalene B (**2**), naphthone C (**3**), daldinone C (**4**) and 8-methoxynaphthalene-1,7-diol (**7**) showed anti-acetylcholinesterase activities.

## Figures and Tables

**Table 1:** ^1^H (500 MHz) and ^13^C-NMR (125 MHz) Data of Compounds **1**–**2** (in CDCl_3_).

No.	1			2
	*δ*_C_	*δ*_H_ (*J* in Hz)	*δ*_C_	*δ*_H_ (*J* in Hz)
1	69.7	4.85 dd (5.4, 8.8)	67.5	4.87 t (2.7)
2*α*	29.2	1.75 m	26.8	1.81 m
2*β*		1.95 m		1.75 m
3*α*	26.2	2.12 m	23.6	2.37 m
3*β*		2.21 m		1.85 m
4	33.6	4.74 m	33.9	5.03 d (5.4)
4a	127.8		134.2	
5	156.2		157.3	
6	109.9	6.74 d (8.0)	103.9	6.85 d (8.0)
7	127.4	7.27 t (8.0)	125.6	7.45 t (8.0)
8	119.5	7.26 d (8.0)	118.0	7.9 d (8.0)
8a	141.9		140.5	
5-OCH_3_	56.2	4.06 s	55.7	3.49 s
1′	150.4		152.9	
2′	128.1		110.4	6.79 d (8.0)
3′	124.7	7.23 d (8.1)	126.8	6.37 d (8.0)
4′	117.8	7.11 d (8.1)	128.3	
4′a	135.3		131.6	
5′	109.8	6.74 d (7.8)	121.7	7.11 d (7.8)
6′	121.9	7.34 d (7.8)	127.9	7.32 t (7.8)
7′	103.8	6.76 d (7.8)	109.5	6.60 d (7.8)
8′	157.4		157.0	
8′a	115.0		115.7	
8′-OCH_3_	55.7	4.08 s	56.3	3.49 s

**Table 2:** ^1^H (500 MHz) and ^13^C-NMR (125 MHz) Data of Compounds **3** (in CDCl_3_).

No.	3	
	*δ*_C_	*δ*_H_ (*J* in Hz)
1	193.3 (s)	
2	145.3 (d)	7.24 d (9.9)
3	124.6 (d)	6.07 d (9.9)
4	97.5 (s)	
4a	119.4 (s)	
5	157.9 (s)	
6	120.2 (d)	6.94 dd (8.3, 1.0)
7	131.7 (d)	7.27 t (7.5)
8	122.6 (d)	6.83 dd (8.3, 1.0)
8a	132.6 (s)	
OCH_3_	52.3 (q)	3.35 s

**Table 3:** The inhibitory activity of compounds **1**–**15** against AChE.

Compound	Inhibition Rate (%)	Initial Screening Concentration (Final Concentration)/μM
	21.35 ± 0.57	143
2	42.74 ± 0.93	143
3	44.63 ± 0.52	227
4	39.50 ± 2.14	149
5	12.40 ± 0.60	266
6	24.74 ± 1.70	287
7	51.49 ± 0.32	263
8	13.72 ± 1.52	266
9	14.51 ± 5.20	281
10	17.69 ± 0.89	260
11	14.87 ± 3.14	258
12	13.33 ± 1.46	258
13	15.18 ± 2.91	258
14	10.59 ± 3.97	260
15	11.80 ± 4.10	238
Tacrine	71.79 ± 1.11	0.33

**Table 4:** Cytotoxic activities of compounds **1–15** against K562 and SGC-7901 cell lines (IC_50_, mM).

Compound	K562	SGC-7901
1	>0.25	>0.25
2	>0.25	>0.25
3	>0.25	>0.25
4	>0.25	>0.25
5	0.10	0.13
6	>0.25	>0.25
7	0.076	0.18
8	0.058	0.15
9	>0.25	>0.25
10	>0.25	>0.25
11	>0.25	>0.25
12	>0.25	>0.25
13	>0.25	>0.25
14	>0.25	>0.25
15	>0.25	>0.25
taxol	0.00021	0.0010

## Supplementary Materials

The following are available online. Figure S1: ^1^H-NMR spectrum of dipolynaphthalene A (1); Figure S2: ^13^C-NMR spectrum of dipolynaphthalene A (1); Figure S3: HSQC spectrum ofdipolynaphthalene A (1); Figure S4: HMBC spectrum ofdipolynaphthalene A (1); Figure S5: ^1^H-^1^H COSY spectrum of dipolynaphthalene A (1); Figure S6: ROESY spectrum of dipolynaphthalene A (1); Figure S7: HR-ESI-MS of dipolynaphthalene A (1); Figure S8: IR spectrum of dipolynaphthalene A (1); Figure S9: ^1^H-NMR spectrum of dipolynaphthalene B (2); Figure S10: ^13^C-NMR spectrum of dipolynaphthalene B (2); Figure S11: HSQC spectrum ofxylariaine B (2); Figure S12: HMBC spectrum of dipolynaphthalene B (2); Figure S13: ^1^H-^1^H COSY spectrum of dipolynaphthalene B (2); Figure S14: ROESY spectrum of dipolynaphthalene B (2); Figure S15: HR-ESI-MS of dipolynaphthalene B (2); Figure S16: IR spectrum of dipolynaphthalene B (2); Figure S17: ^1^H-NMR spectrum ofnaphthone C (3); Figure S18: ^13^C-NMR spectrumof naphthone C (3); Figure S19: HSQC spectrum of naphthone C (3); Figure S20: HMBC spectrum of naphthone C (3); Figure S21: ^1^H-^1^H COSY spectrum ofnaphthone C (3); Figure S22: HR-ESI-MS of naphthone C (3); Figure S23: IR spectrum of naphthone C (3).

## References

[1] Liu R.X., Chang P., Jiang Z.F. (2014). Research Progress of Bioactive Polysaccharides in Neuroprotection and in the Prevention and Treatment of Alzheimer’s Disease. Nat. Prod. Res. Dev..

[2] Hiremathad A., Piemontese L. (2017). Heterocyclic compounds as key structures for the interaction with old and new targets in Alzheimer’s disease therapy. Neural Regen. Res..

[3] Song B., Deng C.Y., Wu X.L., Li T.H. (2009). Known Species of *Marasmius* from China and Their Distribution. Guizhou Sci..

[4] Ayer W.A., Craw P.A., Stout T.J., Clardy J. (1989). Novel sesquiterpenoids from the fairy ring fungus, *Marasmius oreades*. Can. J. Chem..

[5] Evans L., Hedger J., O’Donnell G., Skelton B.W., White A.H., Williamson R.T., Gibbons S. (2010). Structure elucidation of some highly unusual tricyclic cis-caryophyllane sesquiterpenes from *Marasmiellus troyanus*. Tetrahedron Lett..

[6] Meng J., Li Y.Y., Ou Y.X., Song L.F., Lu C.H., Shen Y.M. (2011). New sesquiterpenes from *Marasmius cladophyllus*. Mycology.

[7] Liermann J.C., Thines E., Opatz T., Anke H. (2012). Drimane sesquiterpenoids from *Marasmius* sp. inhibiting the conidial germination of plant-pathogenic fungi. J. Nat. Prod..

[8] Isaka M., Palasarn S., Sappan M., Supothina S., Boonpratuang T. (2016). Hirsutane sesquiterpenes from cultures of the *basidiomycete Marasmiellus* sp. BCC 22389. Nat. Prod. Bioprospect..

[9] Fattorusso E., Giovannitti B., Lanzotti V., Magno S., Violante U. (1992). 4,4-Dimethyl-5 alpha -ergosta -8,24(28)-dien-3 beta-ol from the fungus *Marasmius oreades*. Steroids.

[10] Ványolós A., Dékány M., Kovács B., Krámos B., Bérdi P., Zupkó I., Hohmann J., Béni Z. (2016). Gymnopeptides A and B, cyclic octadecapeptides from the mushroom *Gymnopus fusipes*. Org. Lett..

[11] Thongbai B., Surup F., Mohr K., Kuhnert E., Hyde K.D., Stadler M. (2013). Gymnopalynes A and B, chloropropynyl-isocoumarin antibiotics from cultures of the *basidiomycete Gymnopus* sp. J. Nat. Prod..

[12] Zhang L., Yang M., Song Y., Sun Z., Peng Y., Qu K., Zhu H. (2009). Antihypertensive effect of 3,3,5,5-tetramethyl-4-piperidone, a new compound extracted from *Marasmius androsaceus*. J. Ethnopharmacol..

[13] Mao X.L. (2009). Macromycetes of China.

[14] Dai J., Karsten K., Ulrich F., Siegfried D., Barbara S., Attila K.S., Sándor A., Tibor K., Teunis van R. (2006). Metabolites from the endophytic fungus *Nodulisporium* sp. from *Juniperus cedre*. Eur. J. Org. Chem..

[15] Crouse D.J., Wheeler D.M.S. (1979). Preparation of a 4-monoketal of juglone methyl ether. Tetrahedron Lett..

[16] Gu W., Ge H.M., Song Y.C., Ding H., Zhu H.L., Zhao X.A., Tan R.X. (2007). Cytotoxic benzo[*j*]fluoranthene metabolites from *Hypoxylon truncatum* IFB-18, an endophyte of *Artemisia annua*. J. Nat. Prod..

[17] Li D.L., Wu Z.C., Chen Y.C., Tao M.H., Zhang W.M. (2011). Chemical constituents of endophytic fungus *Nodulisporium* sp. A4 from *Aquilaria sinensis*. China J. Chin. Mater. Med..

[18] Nadeau A.K., Sorensen J.L. (2011). Polyketides produced by *Daldinia loculata* cultured from Northern Manitoba. Tetrahedron Lett..

[19] Chang C., Chang H., Cheng M. (2014). Inhibitory effects of constituents of an endophytic fungus *Hypoxylon investiens* on nitric oxide and interleukin-6 production in RAW264.7 macrophages. Chem. Biodivers..

[20] Tietze L.F., Güntner C., Gericke K.M. (2005). A Diels-Alder reaction for the total synthesis of the novel antibiotic antitumor agent mensacarcin. Eur. J. Org. Chem..

[21] William A.A., Latchezar S.T., Leonard J.H. (2000). Metabolites from a wood-inhabiting cup fungus, *Urnula craterium*. Nat. Prod. Lett..

[22] Arai M., Yamamoto K., Namatame I. (2003). New monordens produced by amidepsine-producing fungus *Humicola* sp. FO-2942. J. Antibiot..

[23] Dong J.Y., Song H.C., Li J.H. (2008). Ymf 1029A-E, preussomerin analogues from the fresh-water-derived fungus YMF 1.01029. J. Nat. Prod..

[24] Couché E., Fkyerat A., Tabacchi R. (2010). Stereoselective synthesis of *cis*- and *trans*-3,4-dihydro-3, 4,8-trihydroxynaphthalen-1(2*H*)-one. Helv. Chim. Acta.

[25] Li X.J., Gao J.M., Zhang A.L. (2012). Toxins from a symbiotic fungus, *Leptographium qinlingensis* associated with *Dendroctonus armandi* and their in vitro toxicities to *Pinus armandi* seedlings. Eur. J. Plant Pathol..

[26] Rukachaisirikul V., Sommart U., Phongpaichit S. (2007). Metabolites from the xylariaceous fungus PSU-A80. Chem. Pharm. Bull..

[27] Huang R., Wang T., Xie X.S. (2016). Secondary metabolites from an endophytic fungus *Nigrospora* sp. Chem. Nat. Compd..

[28] Ellman G.L., Courtney K.D., Andres V., Featherstone R.M. (1961). A new and rapid colorimetric determination of acetylcholinesterase activity. Biochem. Pharmacol..

[29] Mosmann T. (1983). Rapid colorimetric assay for cellular growth and survival: Application to proliferation and cytotoxicity assays. J. Immunol. Methods.

[30] Barnes E.C., Jumpathong J., Lumyong S., Voigt K., Hertweck C. (2016). Daldionin, an unprecedented binaphthyl derivative, and diverse polyketide congeners from a fungal orchid endophyte. Chem. Eur. J..

