# Supplementary material for: Napthrene Compounds from Mycelial Fermentation Products of Marasmius berteroi
Source: Molecules. 2020 Aug 26;25(17):3898. doi: 10.3390/molecules25173898 (PMC7504358; doi:10.3390/molecules25173898)

Napthrene Compounds from Mycelial Fermentation Products of *Marasmius berteroi*

Ning Ning Yang[1,4, ⊥], Qing Yun Ma[1, 3,⊥], Fan Dong Kong[1,3], Qing Yi Xie[1,3], Hao Fu Dai[1,3], Li Man Zhou[1], Zhi Fang Yu[2*], You Xing Zhao[1,3*]

[1]Hainan Key Laboratory for Research and Development of Natural Product from Li Folk Medicine,, Institute of Tropical Bioscience and Biotechnology, Chinese Academy of Tropical Agricultural Sciences, Haikou 571101, China; yangningning891216@163.com (N.N.Y.); maqingyun@itbb.org.cn (Q.Y.M.); kongfandong@itbb.org.cn (F.D.K.); xieqingyi@itbb.org.cn (Q.Y.X.)

[2]College of Food Science and Technology, Nanjing Agricultural University, Nanjing 210095, China; yuzhifang@njau.edu.cn(Z.F.Y.)

[3]Hainan Institute for Tropical Agricultural Resources, Chinese Academy of Tropical Agricultural Sciences, Haikou 571101, China;

[4]School of Food and Biological Engineering, Bengbu University, Bengbu 233030, China

⊥Ning Ning Yang and Qing Yun Ma contributed equally to this work.

*Correspondence: zhaoyouxing@itbb.org.cn (Y.X.Z.);yuzhifang@njau.edu.cn (Z.F.Y.)

Supplementary data

Figure S1. ^{1}H NMR spectrum of dipolynaphthalene A (**1**)

Figure S2. ^{13}C NMR spectrum of dipolynaphthalene A (**1**)

Figure S3. HSQC spectrum of dipolynaphthalene A (**1**)

Figure S4. HMBC spectrum of dipolynaphthalene A (**1**)

Figure S5. ^{1}H-^{1}H COSY spectrum of dipolynaphthalene A (**1**)

Figure S6. ROESY spectrum of dipolynaphthalene A (**1**)

Figure S7. HRESIMS of dipolynaphthalene A (**1**)

Figure S8. IR spectrum of dipolynaphthalene A (**1**)

Figure S9. ^{1}H NMR spectrum of dipolynaphthalene B (**2**)

Figure S10. ^{13}C NMR spectrum of dipolynaphthalene B (**2**)

Figure S11. HSQC spectrum of dipolynaphthalene B (**2**)

Figure S12. HMBC spectrum of dipolynaphthalene B (**2**)

Figure S13. ^{1}H-^{1}H COSY spectrum of dipolynaphthalene B (**2**)

Figure S14. ROESY spectrum of dipolynaphthalene B (**2**)

Figure S15. HRESIMS of dipolynaphthalene B (**2**)

Figure S16. IR spectrum of dipolynaphthalene B (**2**)

Figure S17. ^{1}H NMR spectrum of naphthone C (**3**)

Figure S18. ^{13}C NMR spectrum of naphthone C (**3**)

Figure S19. HSQC spectrum of naphthone C (**3**)

Figure S20. HMBC spectrum of naphthone C (**3**)

Figure S21. ^{1}H-^{1}H COSY spectrum of naphthone C (**3**)

Figure S22. HRESIMS of naphthone C (**3**)

Figure S23. IR spectrum of naphthone C (**3**)

Figure S1. ^{1}H NMR spectrum of dipolynaphthalene A (1)

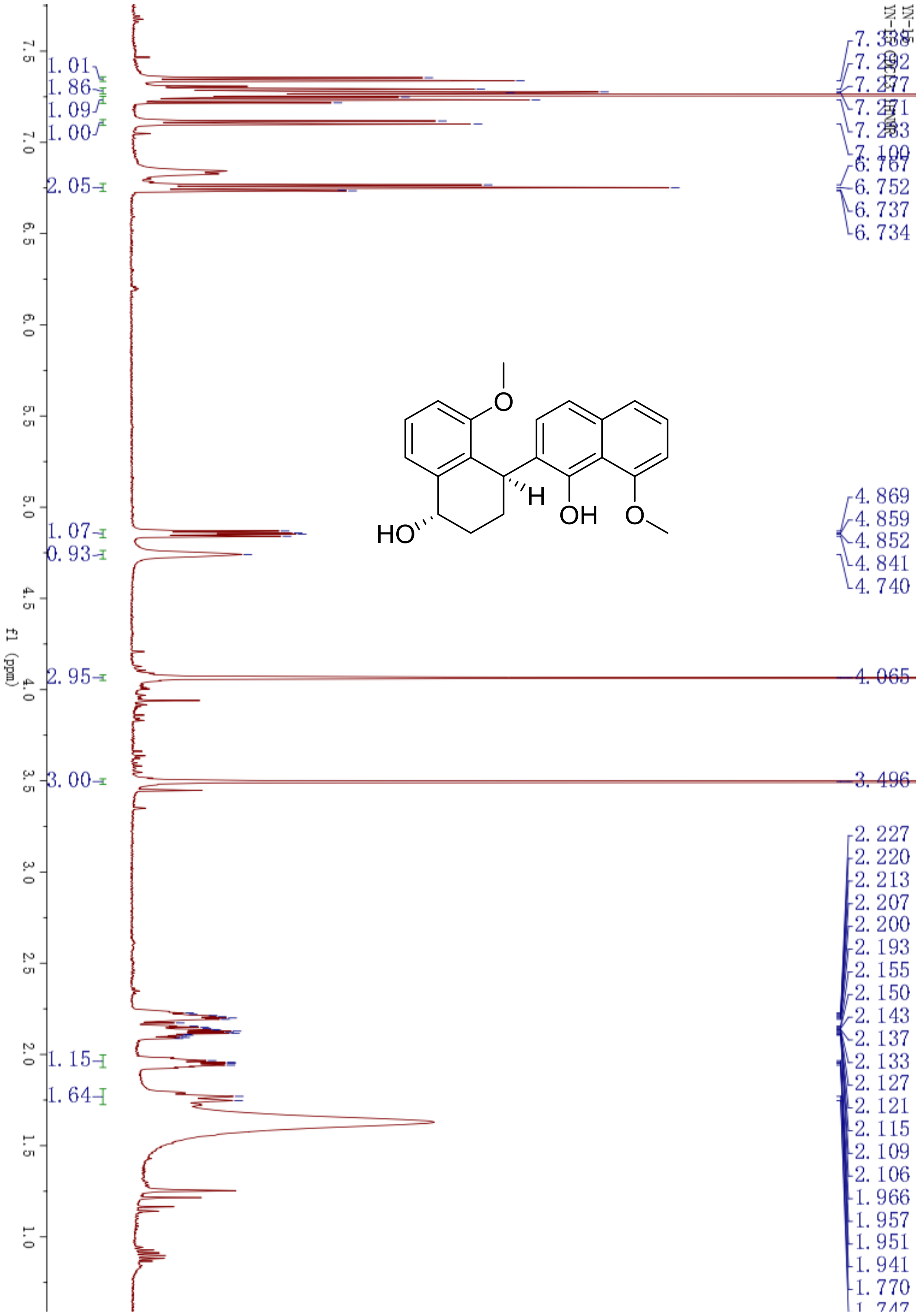

Figure S2. ^{13}C NMR spectrum of dipolynaphthalene A (1)

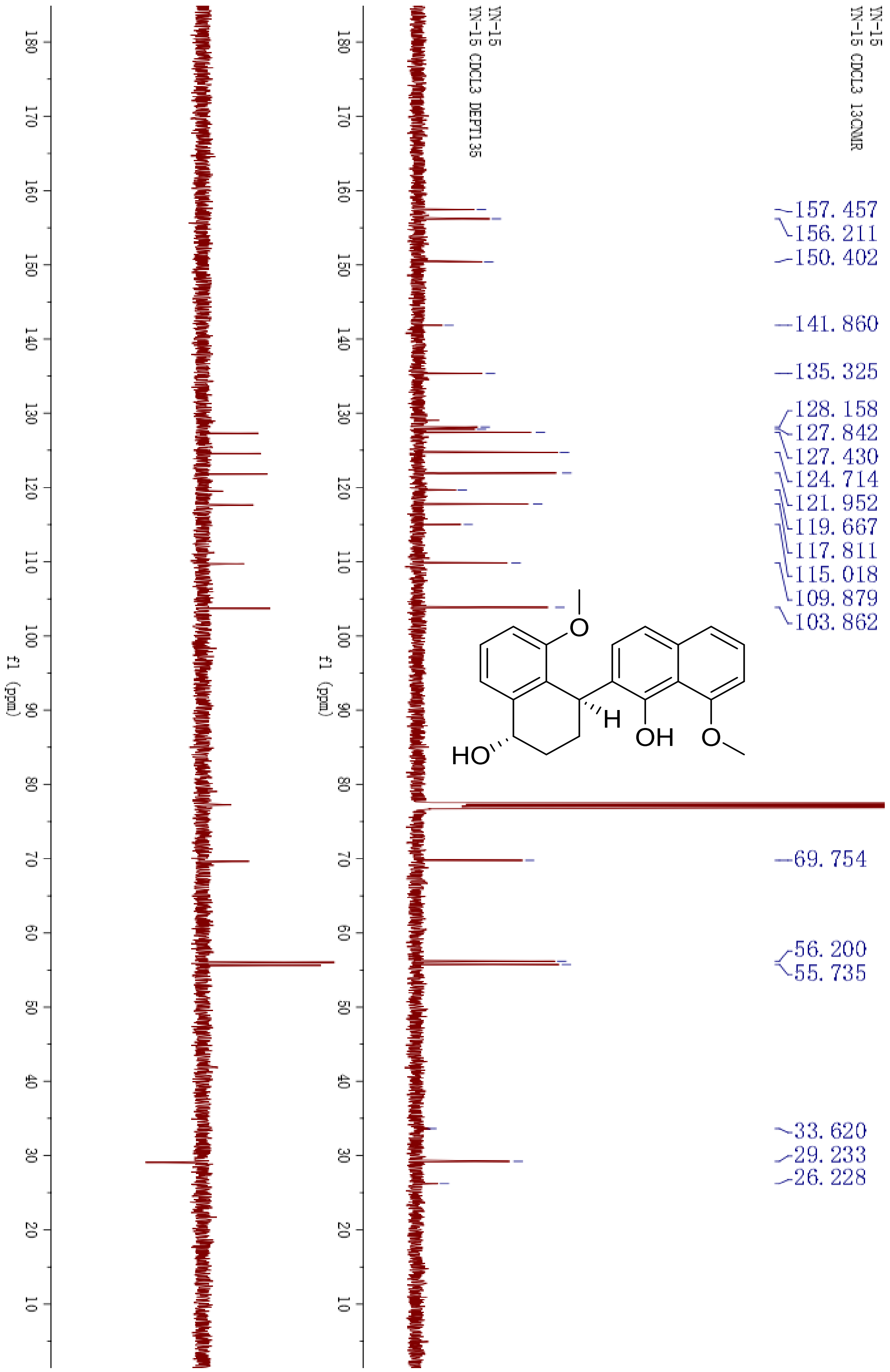

Figure S3. HSQC spectrum of dipolynaphthalene A (1)

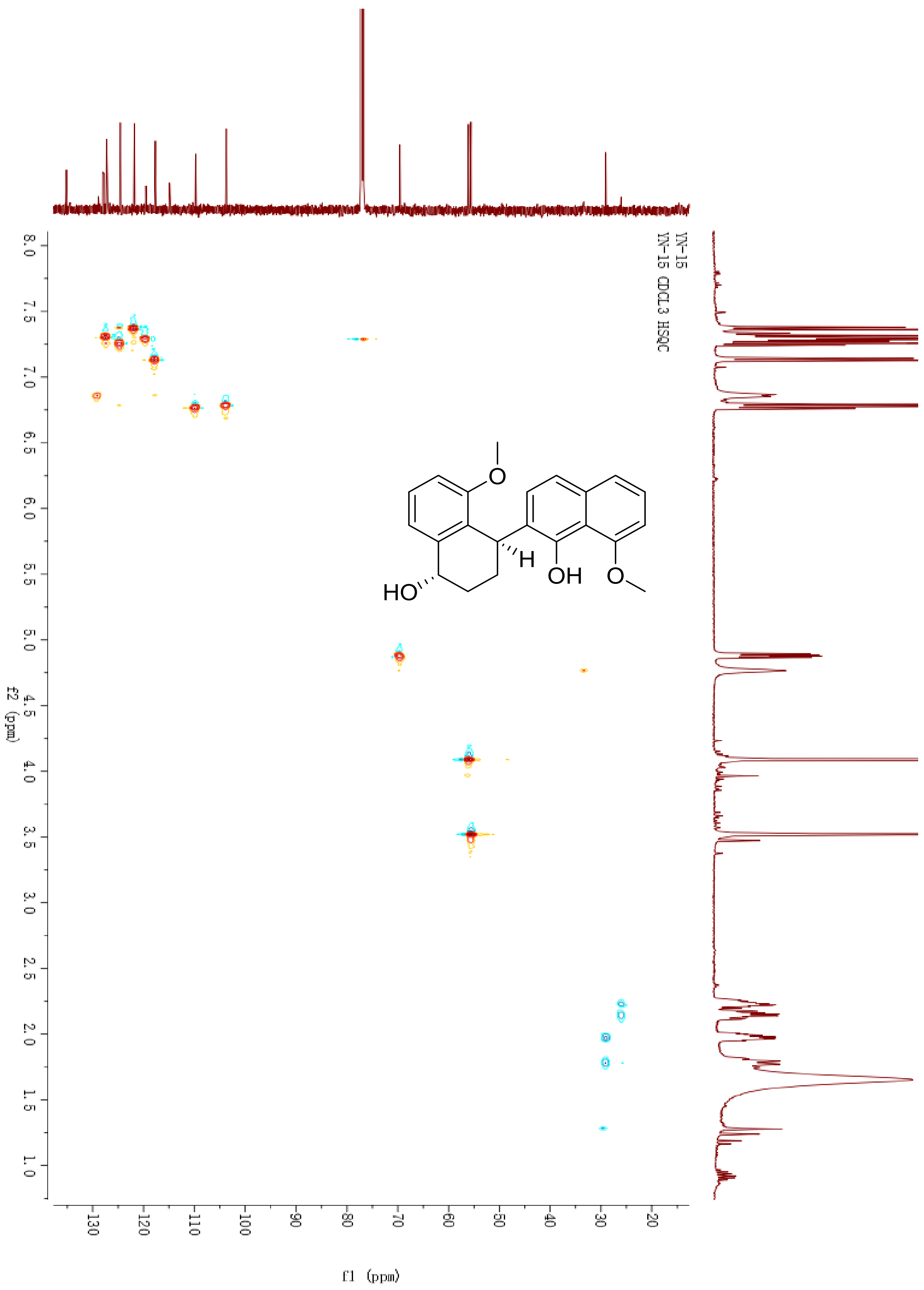

Figure S4. HMBC spectrum of dipolynaphthalene A (1)

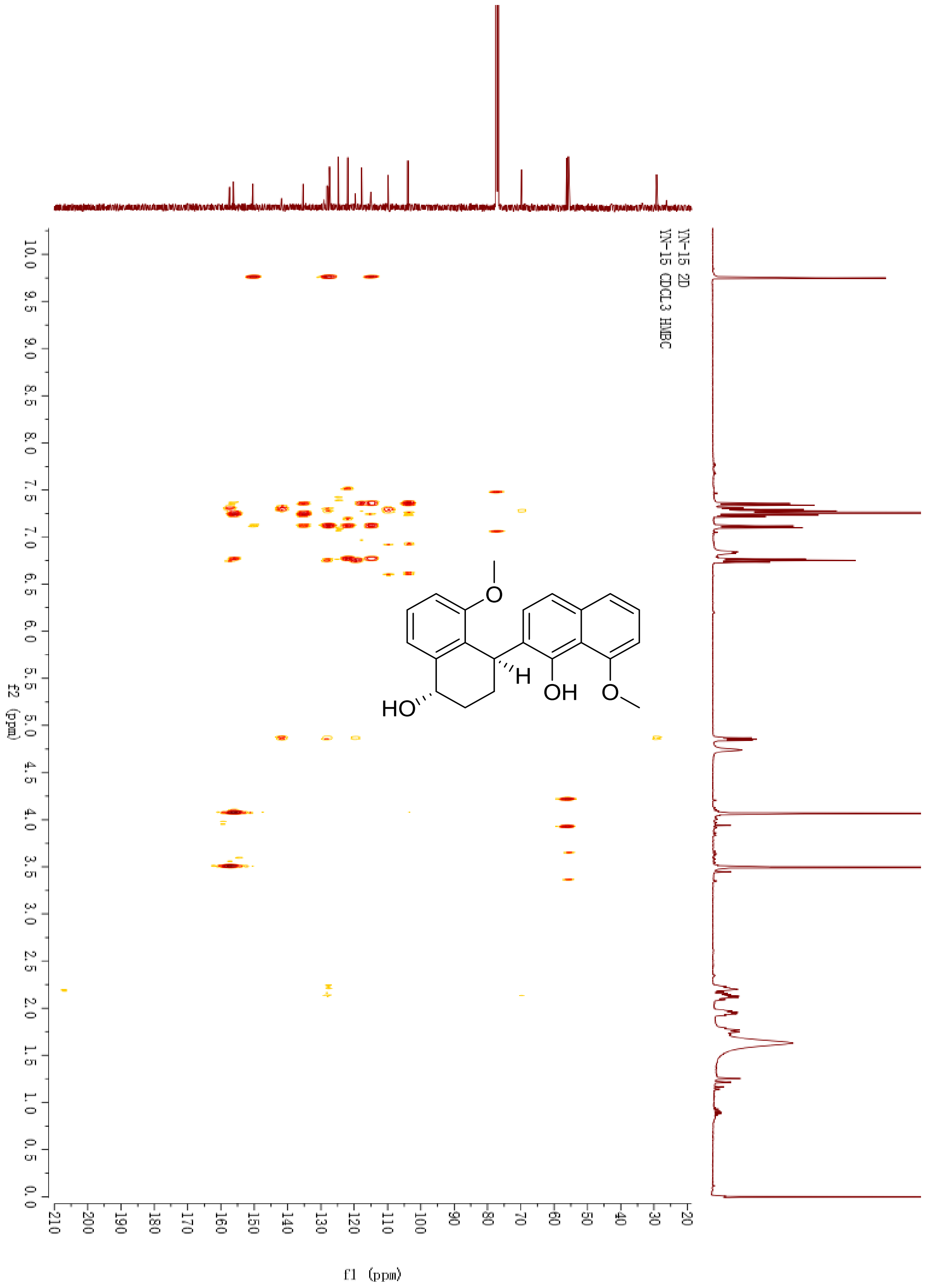

Figure S5. ^{1}H-^{1}H COSY spectrum of dipolynaphthalene A (1)

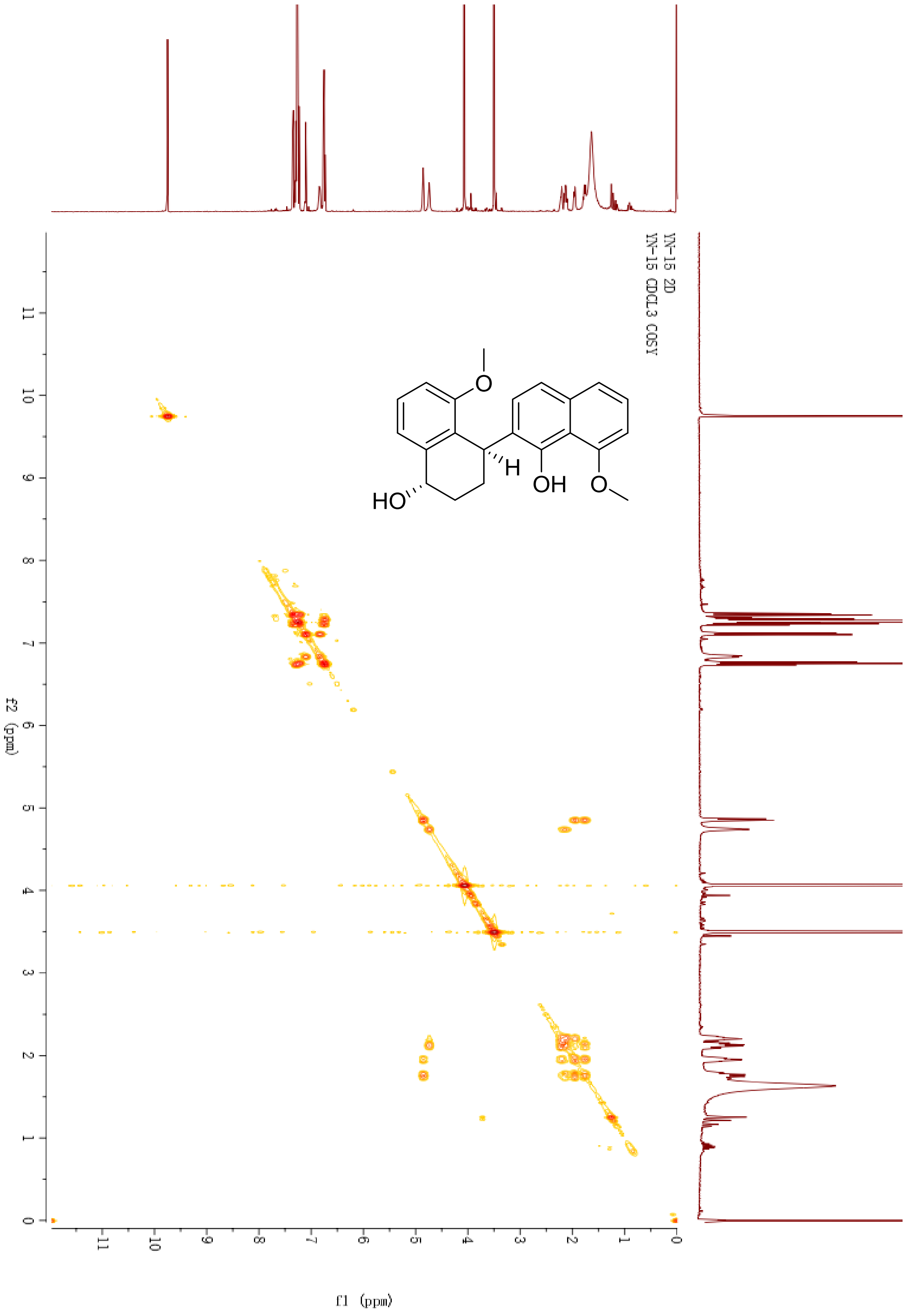

Figure S6. ROESY spectrum of dipolynaphthalene A (1)

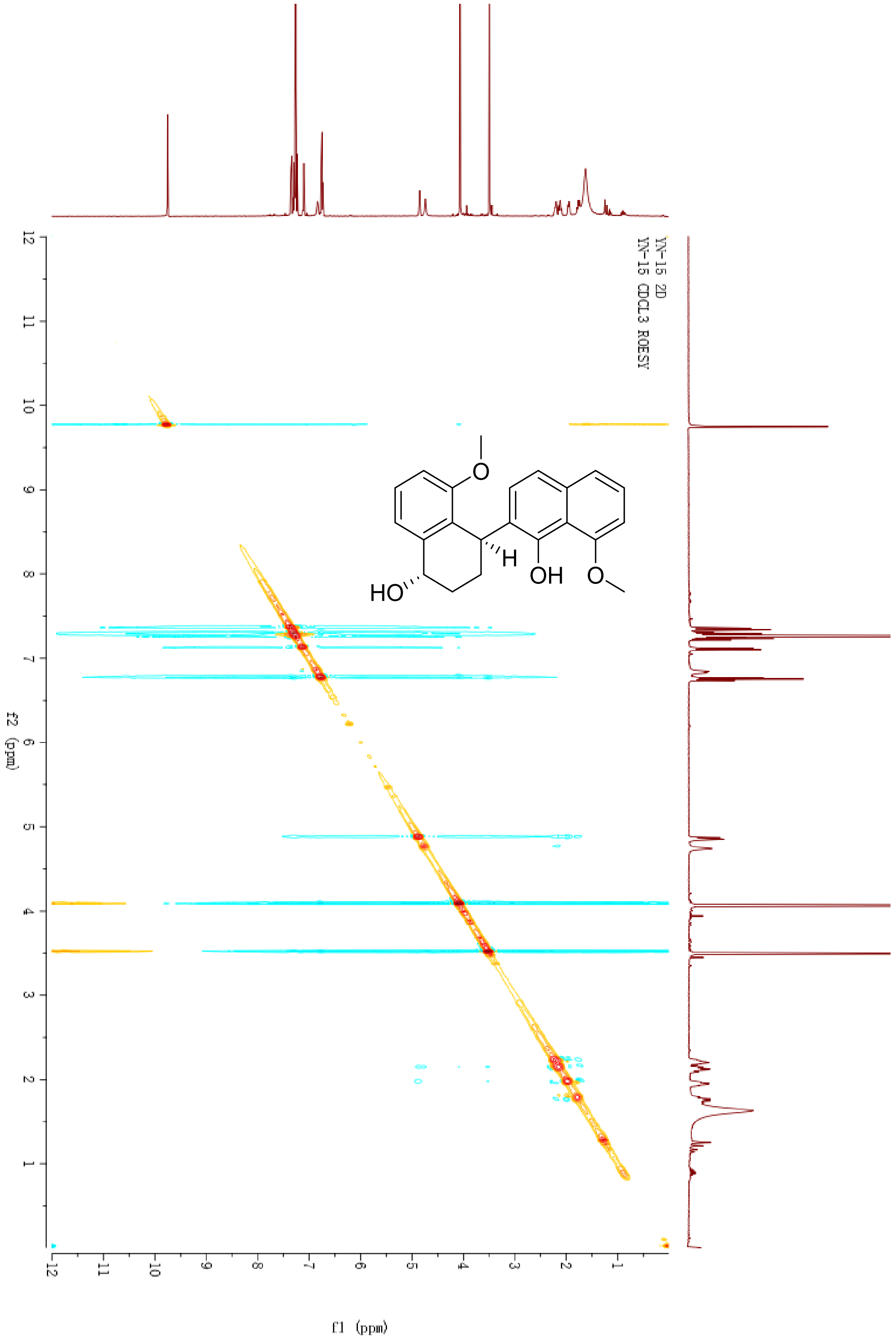

Figure S7. HRESIMS of dipolynaphthalene A (1)

Formula Predictor Report - NY-15.lcd

Page 1 of 1

Data File: E:\DATA\2017\0413\NY-15.lcd

Elmt	Val.	Min	Max	Elmt	Val.	Min	Max	Elmt	Val.	Min	Max	Elmt	Val.	Min	Max	Use Adduct
H	1	0	150	O	2	0	50	S	2	0	0	Pt	2	0	0	Na
B	3	0	0	F	1	0	0	Cl	1	0	0					
C	4	0	100	Na	1	0	0	Br	1	0	0					
N	3	0	10	Si	4	0	0	I	3	0	0					

Error Margin (ppm): 10
HC Ratio: unlimited
Max Isotopes: all
MSn Iso RI (%): 75.00

DBE Range: -2.0 - 100.0
Apply N Rule: yes
Isotope RI (%): 1.00
MSn Logic Mode: AND

Electron Ions: both
Use MSn Info: yes
Isotope Res: 10000
Max Results: 10

Event#: 1 MS(E+) Ret. Time : 0.430 Scan# : 87

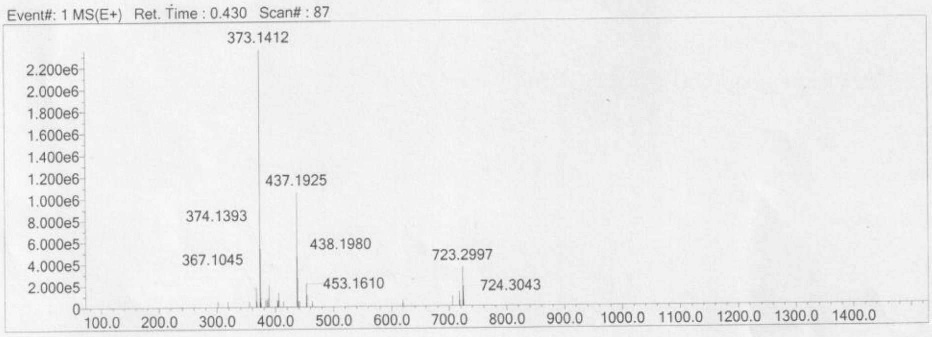

Measured region for 373.1412 m/z

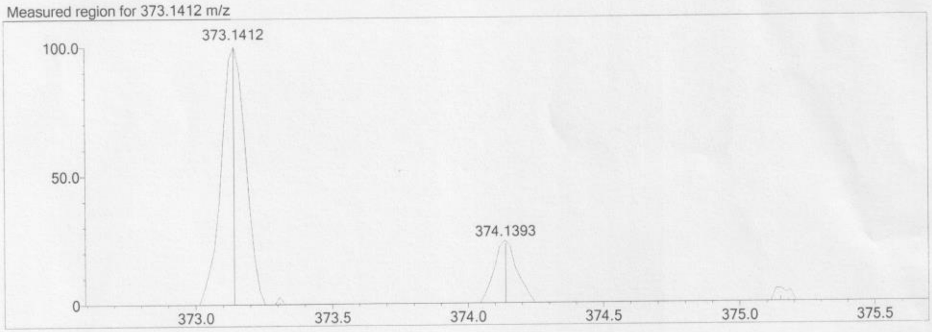

C22 H22 O4 [M+Na]+ : Predicted region for 373.1410 m/z

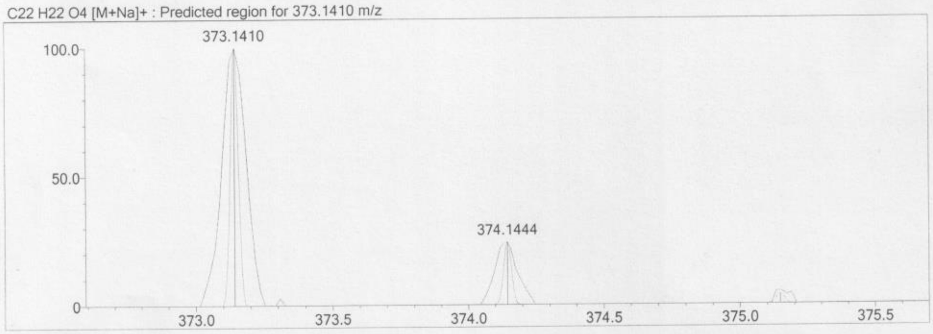

Formula (M)	Ion	Meas. m/z	Pred. m/z	Df. (mDa)	Df. (ppm)	DBE
C22 H22 O4	[M+Na]+	373.1412	373.1410	0.2	0.54	12.0

Figure S8. IR spectrum of dipolynaphthalene A (1)

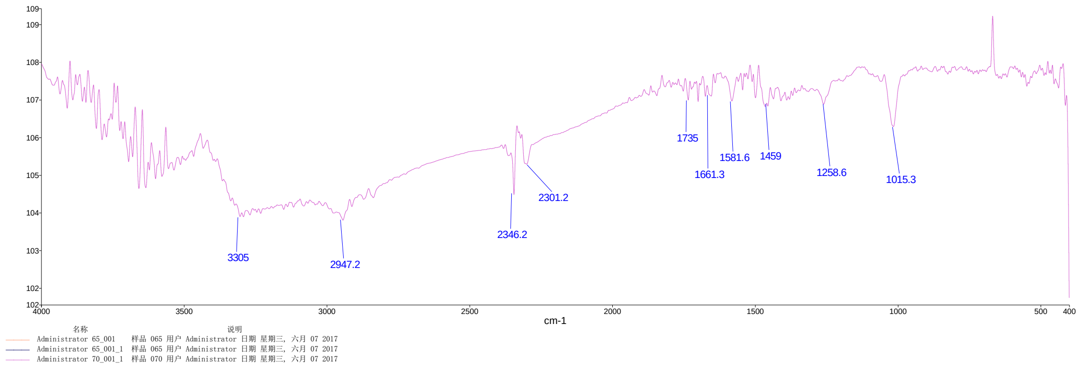

Figure S9. ^{1}H NMR spectrum of dipolynaphthalene B (2)

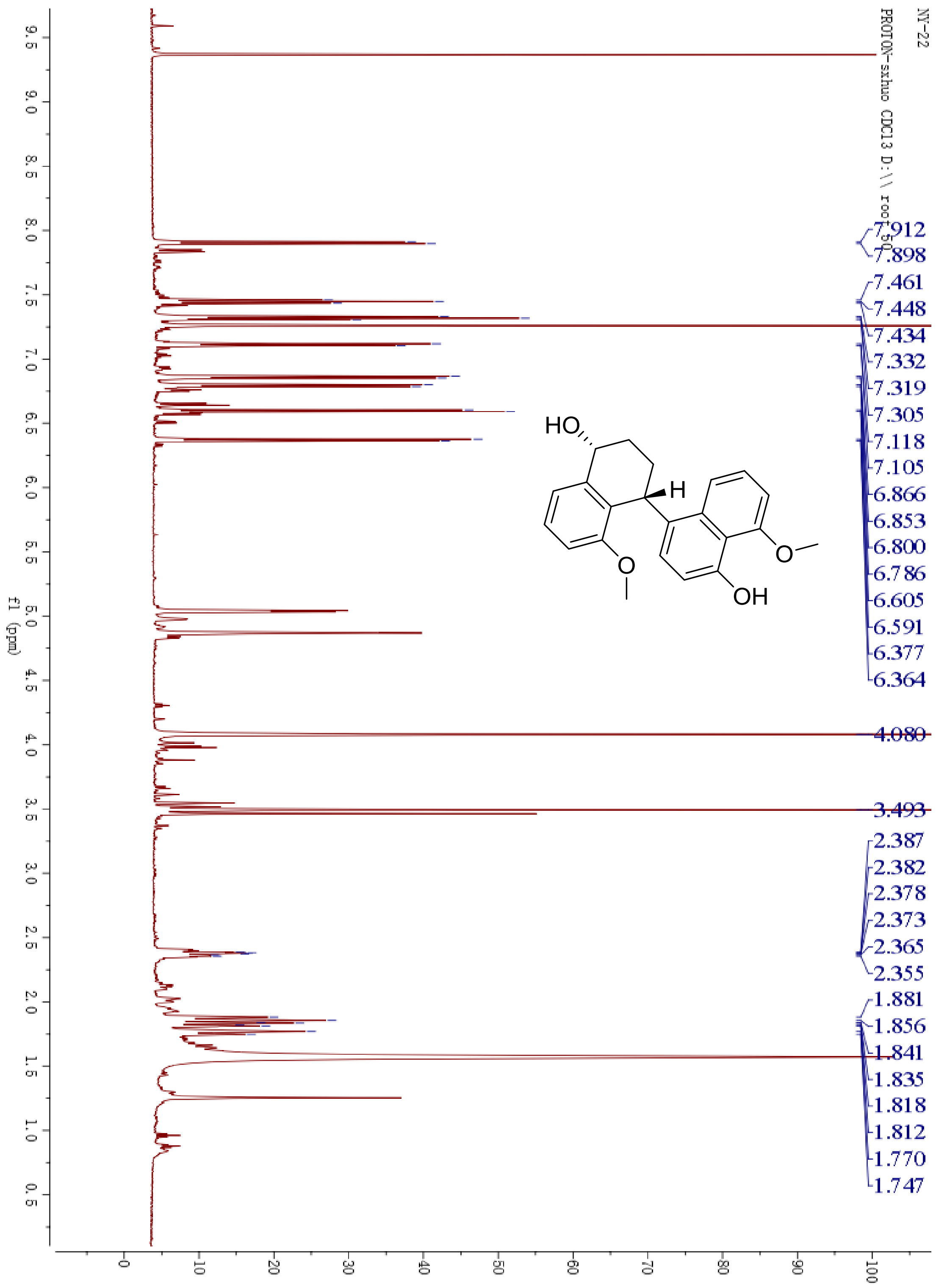

Figure S10. ^{13}C NMR spectrum of dipolynaphthalene B (2)

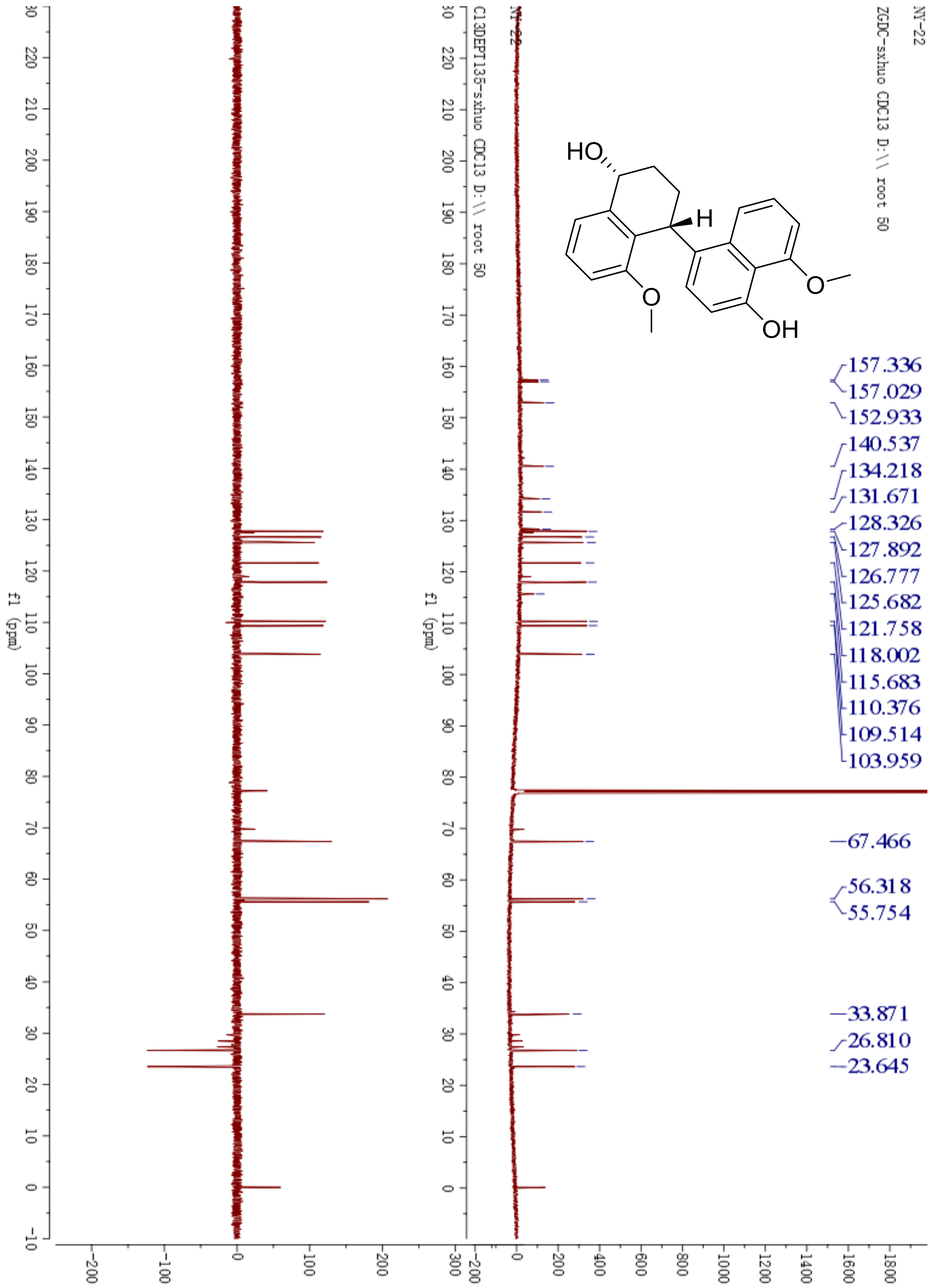

Figure S11. HSQC spectrum of xylariaine B (2)

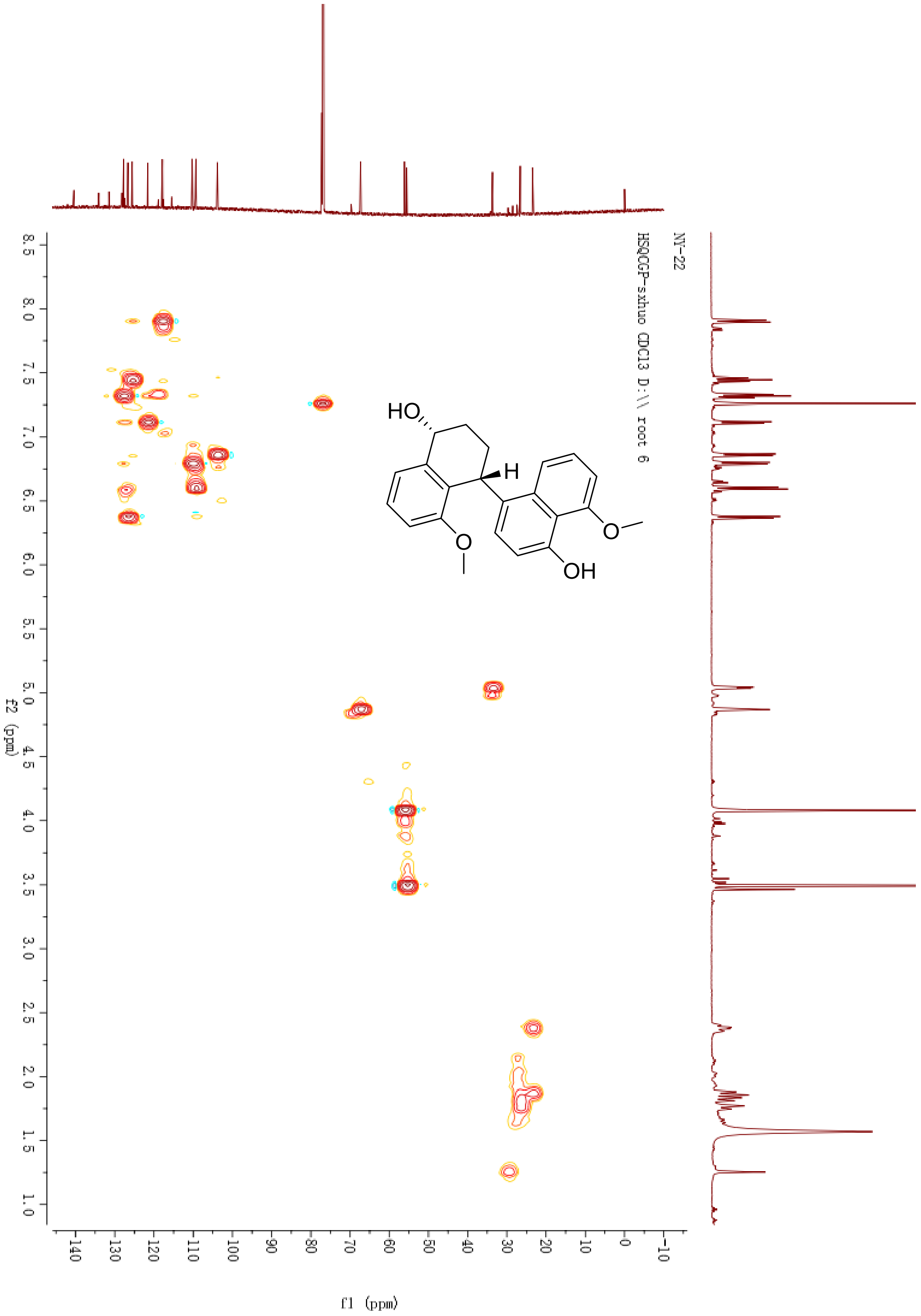

Figure S12. HMBC spectrum of dipolynaphthalene B (2)

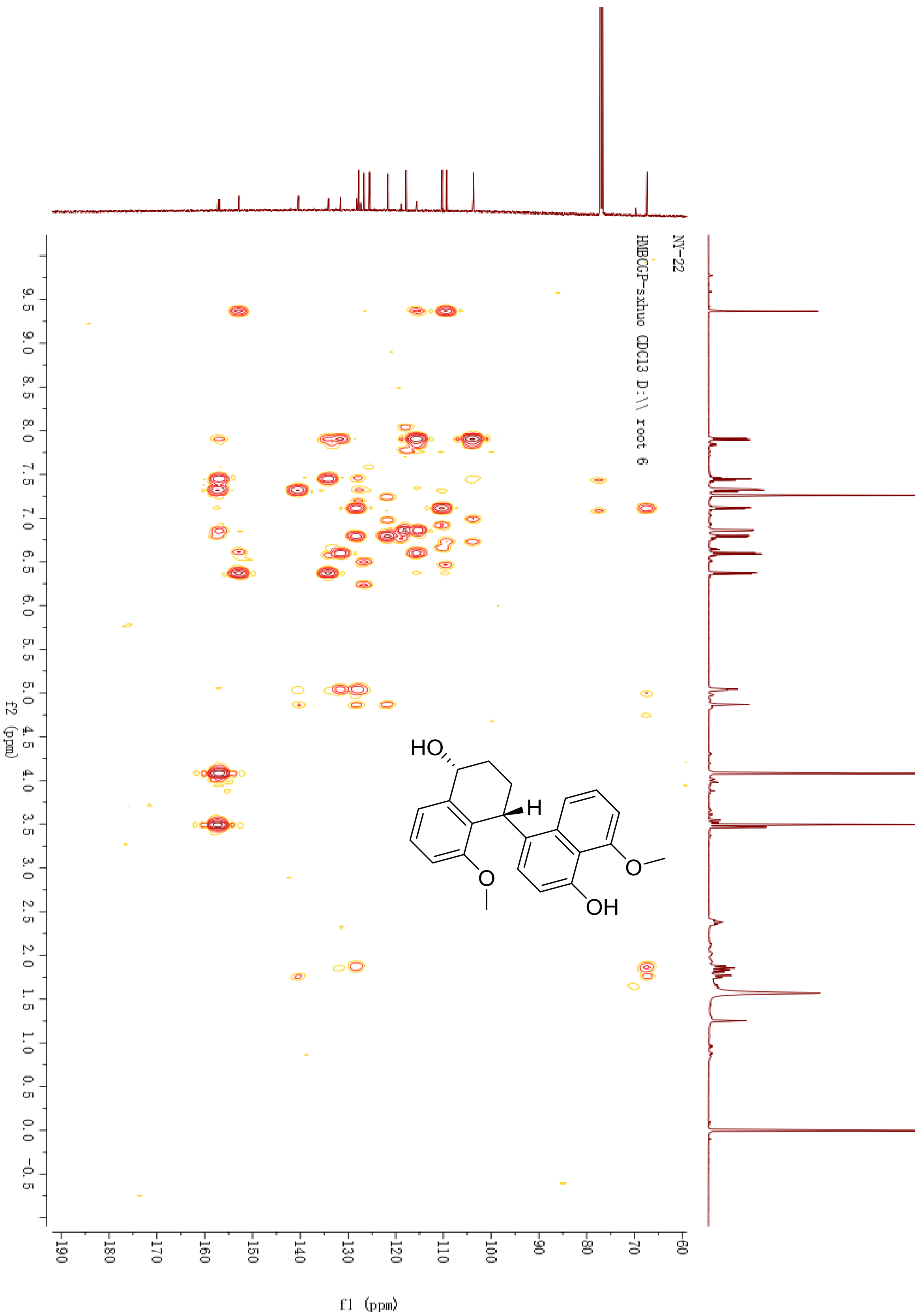

Figure S13. ^{1}H-^{1}H COSY spectrum of dipolynaphthalene B (2)

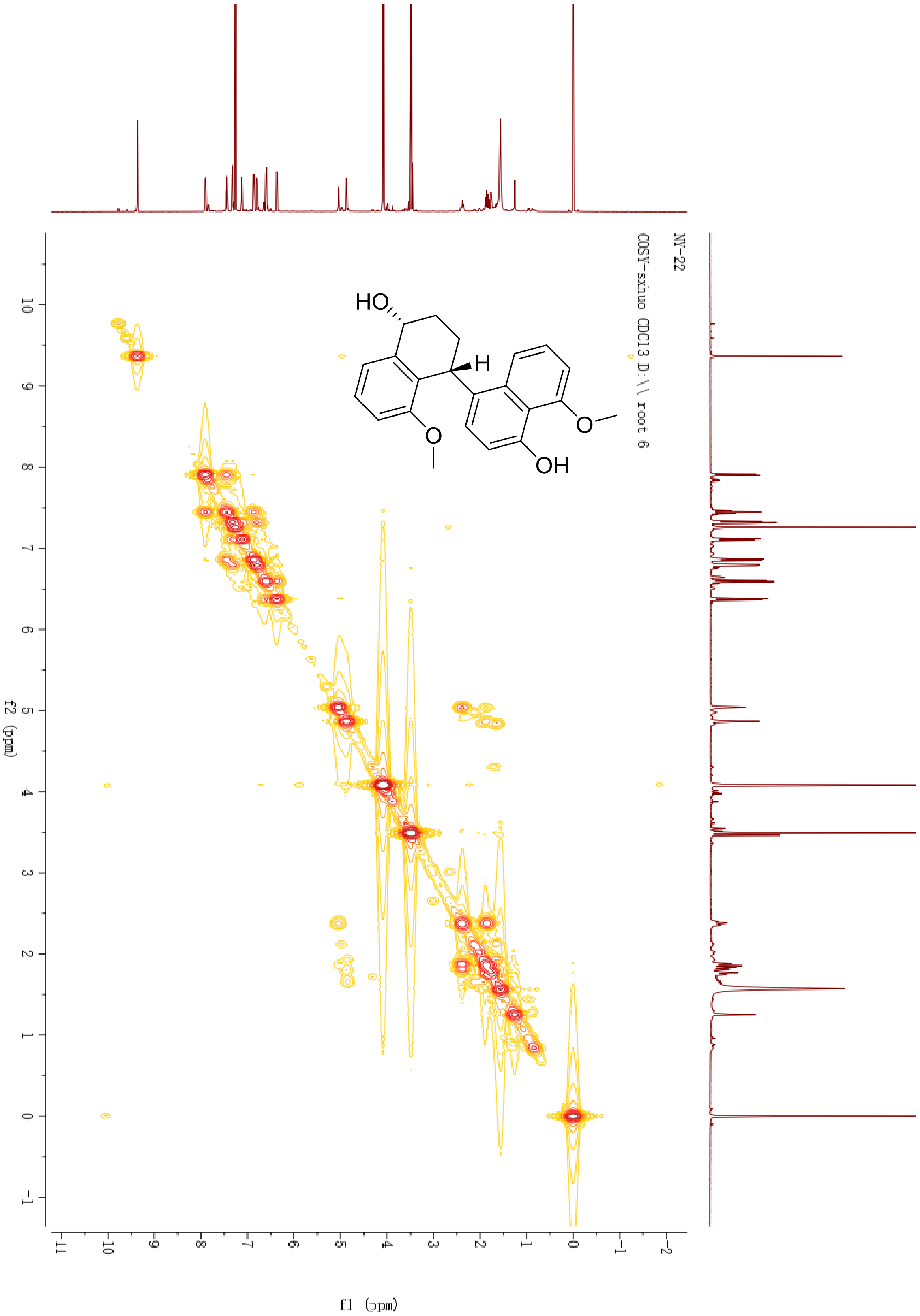

Figure S14. ROESY spectrum of dipolynaphthalene B (2)

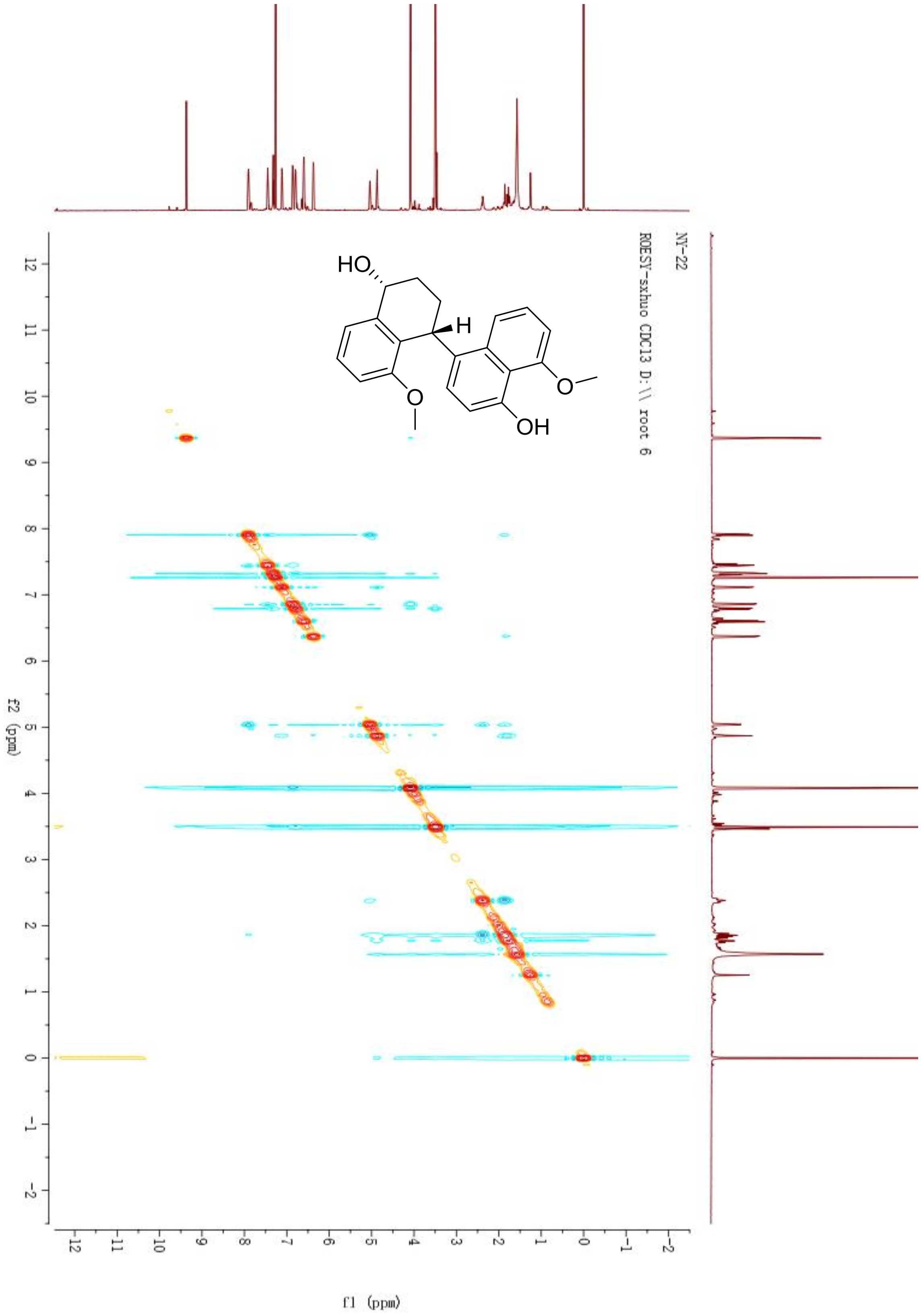

Figure S15. HRESIMS of dipolynaphthalene B (2)

Formula Predictor Report - NY-22.lcd

Data File: E:\DATA\2017\0413\NY-22.lcd

Elmt	Val.	Min	Max	Elmt	Val.	Min	Max	Elmt	Val.	Min	Max	Elmt	Val.	Min	Max	Use Adduct
H	1	0	150	O	2	0	50	S	2	0	0	Pt	2	0	0	Na
B	3	0	0	F	1	0	0	Cl	1	0	0					
C	4	0	100	Na	1	0	0	Br	1	0	0					
N	3	0	10	Si	4	0	0	I	3	0	0					

Error Margin (ppm): 10
HC Ratio: unlimited
Max Isotopes: all
MSn Iso RI (%): 75.00

DBE Range: -2.0 - 100.0
Apply N Rule: yes
Isotope RI (%): 1.00
MSn Logic Mode: AND

Electron Ions: both
Use MSn Info: yes
Isotope Res: 10000
Max Results: 10

Event#: 1 MS(E+) Ret. Time : 0.330 -> 0.390 Scan# : 67 -> 79

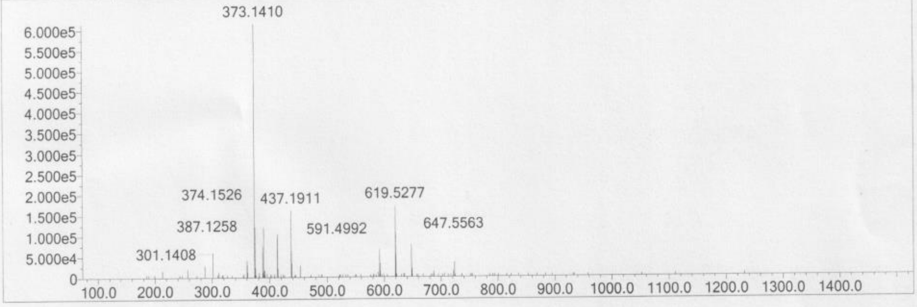

Measured region for 373.1410 m/z

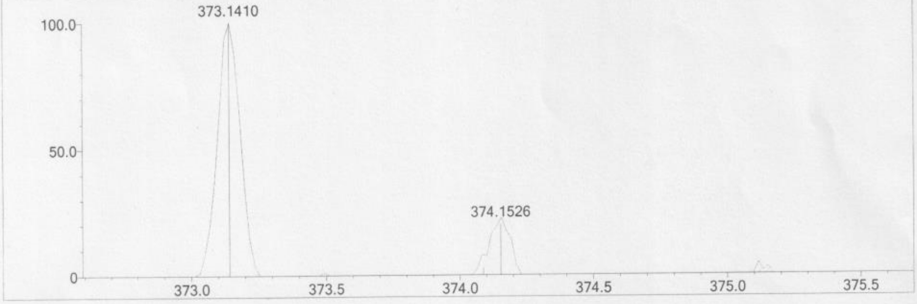

C22 H22 O4 [M+Na]+ : Predicted region for 373.1410 m/z

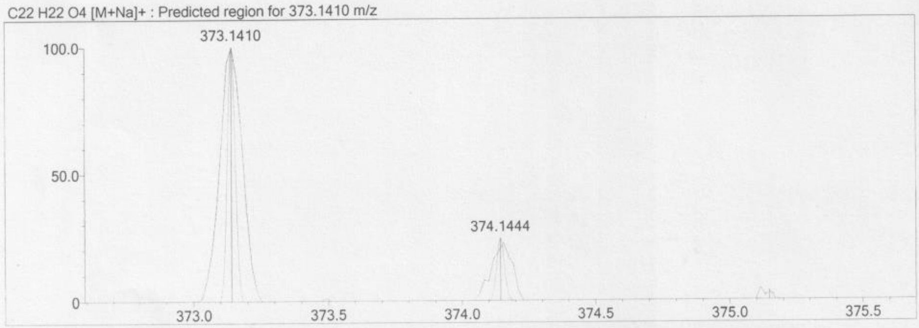

Formula (M)	Ion	Meas. m/z	Pred. m/z	Df. (mDa)	Df. (ppm)	DBE
C22 H22 O4	[M+Na]+	373.1410	373.1410	-0.0	0.00	12.0

Figure S16. IR spectrum of dipolynaphthalene B (2)

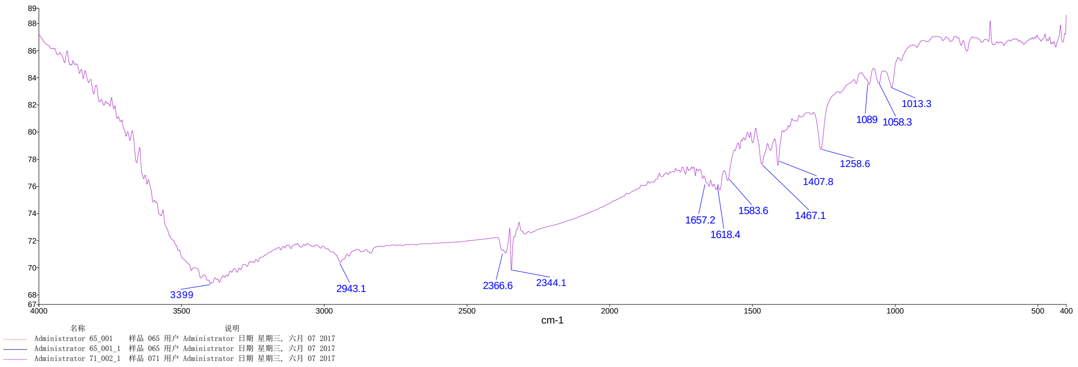

Figure S17. ^{1}H NMR spectrum of naphthone C (3)

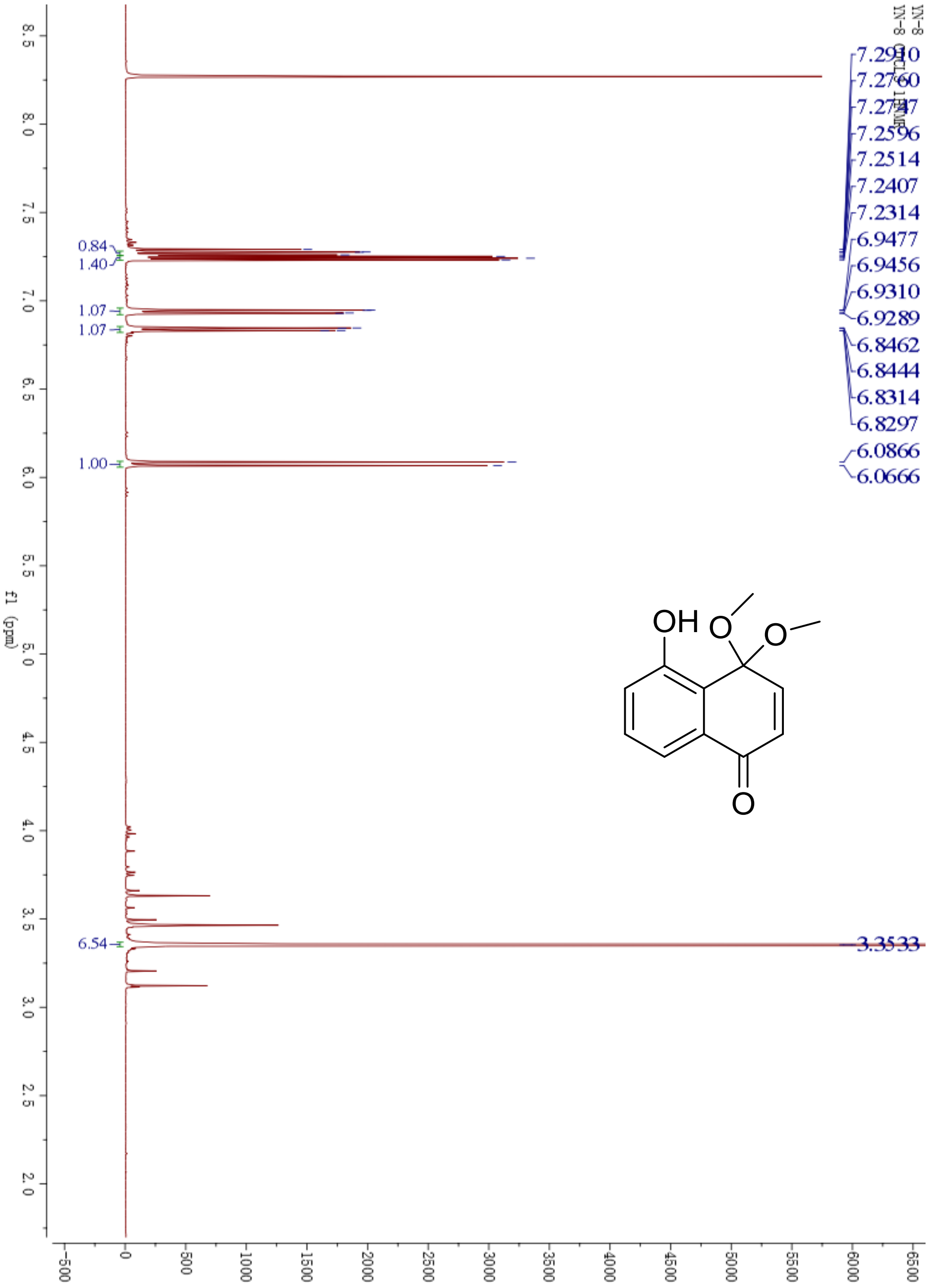

Figure S18. ^{13}C **NMR spectrum of naphthone C (3)**

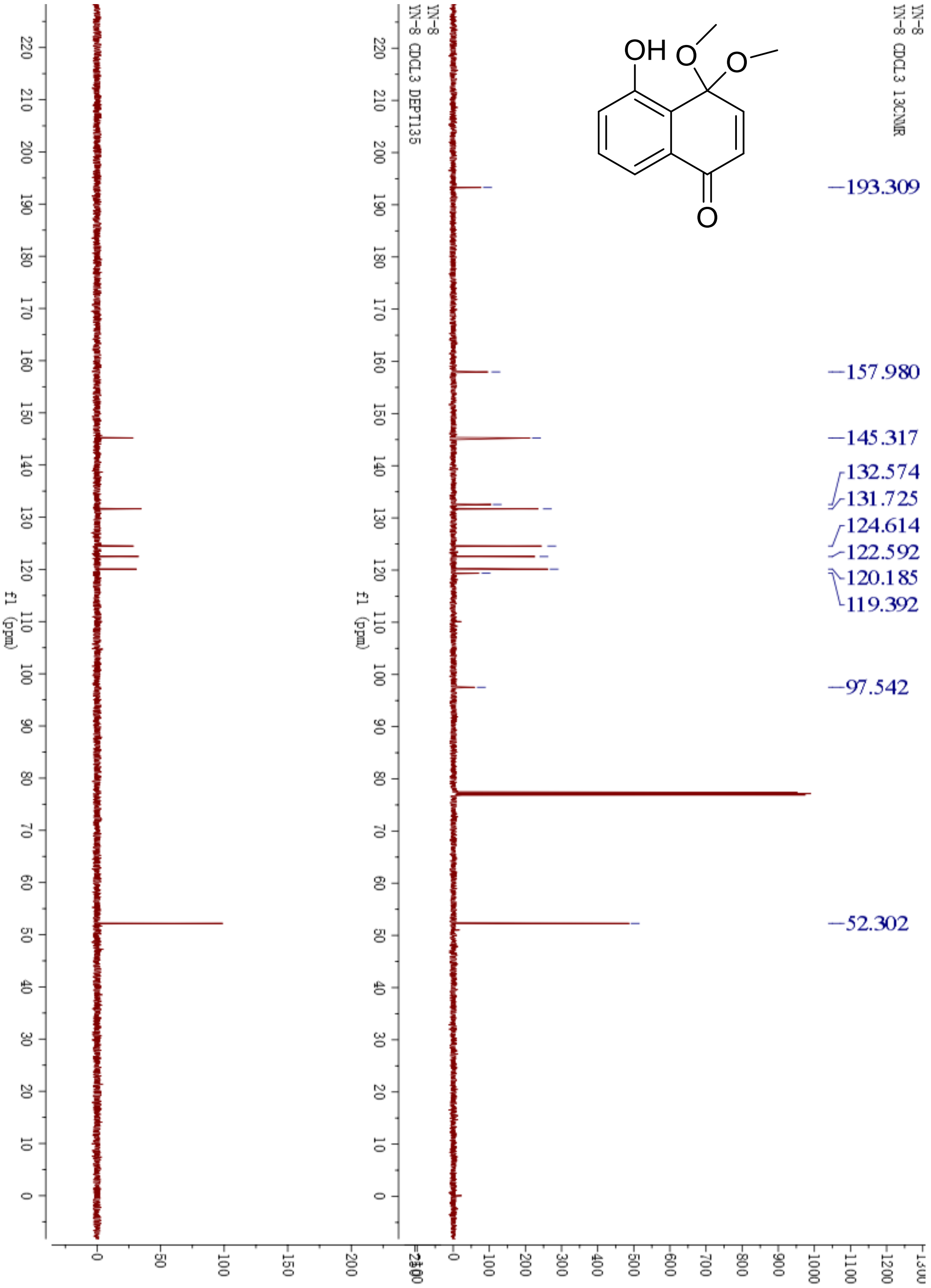

Figure S19. HSQC spectrum of naphthone C (3)

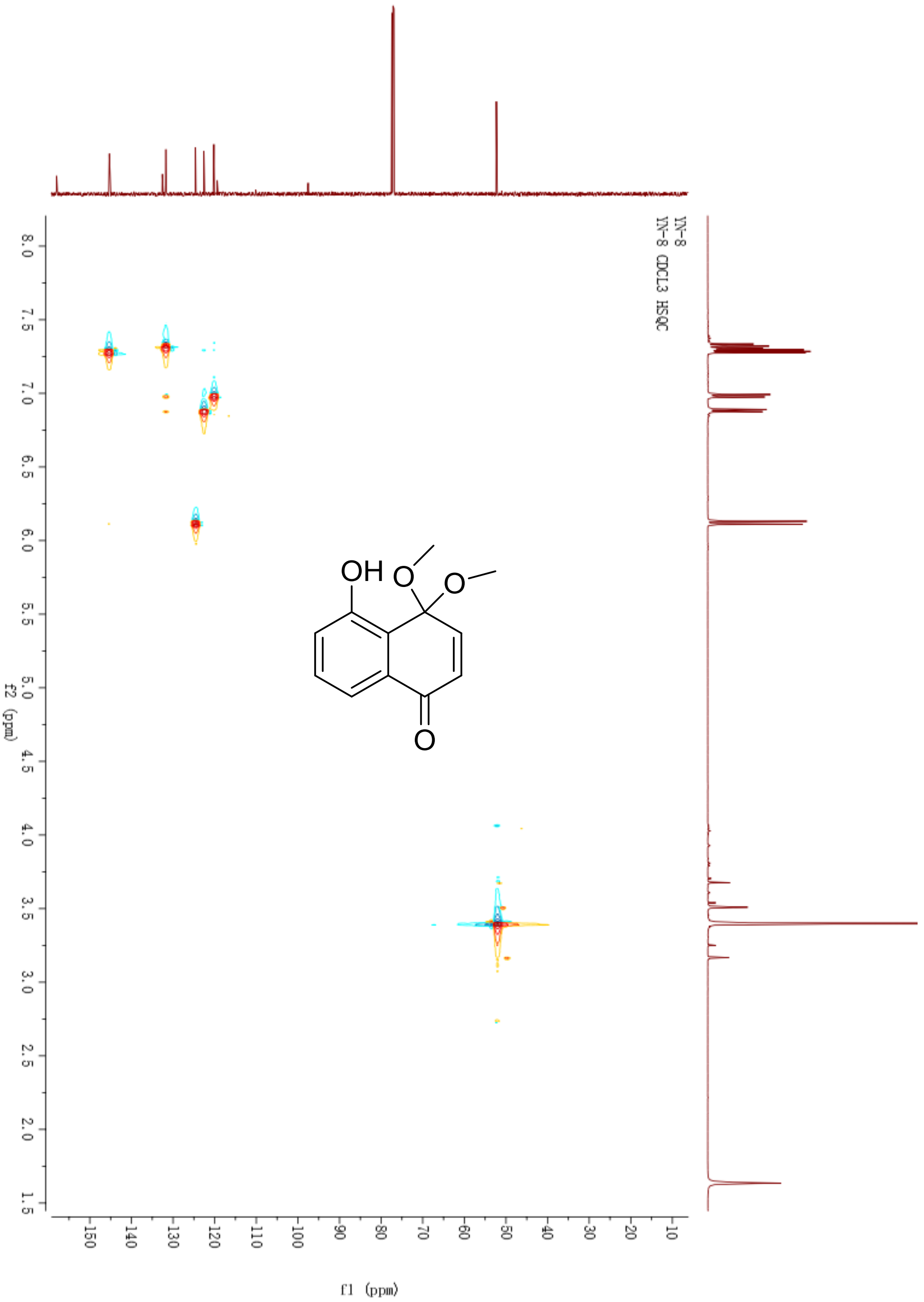

Figure S20. HMBC spectrum of naphthone C (3)

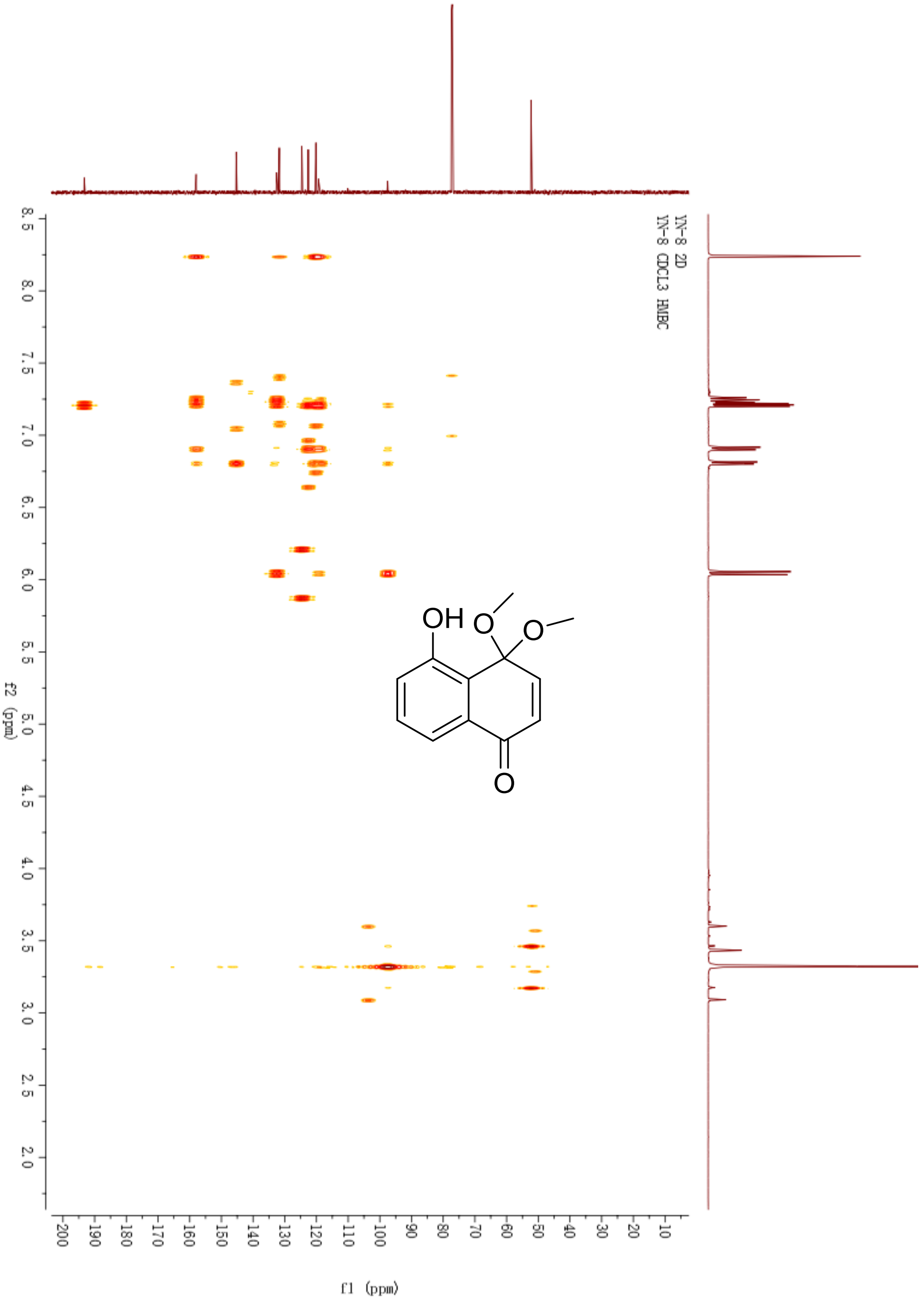

Figure S21. ^{1}H-^{1}H COSY spectrum of naphthone C (3)

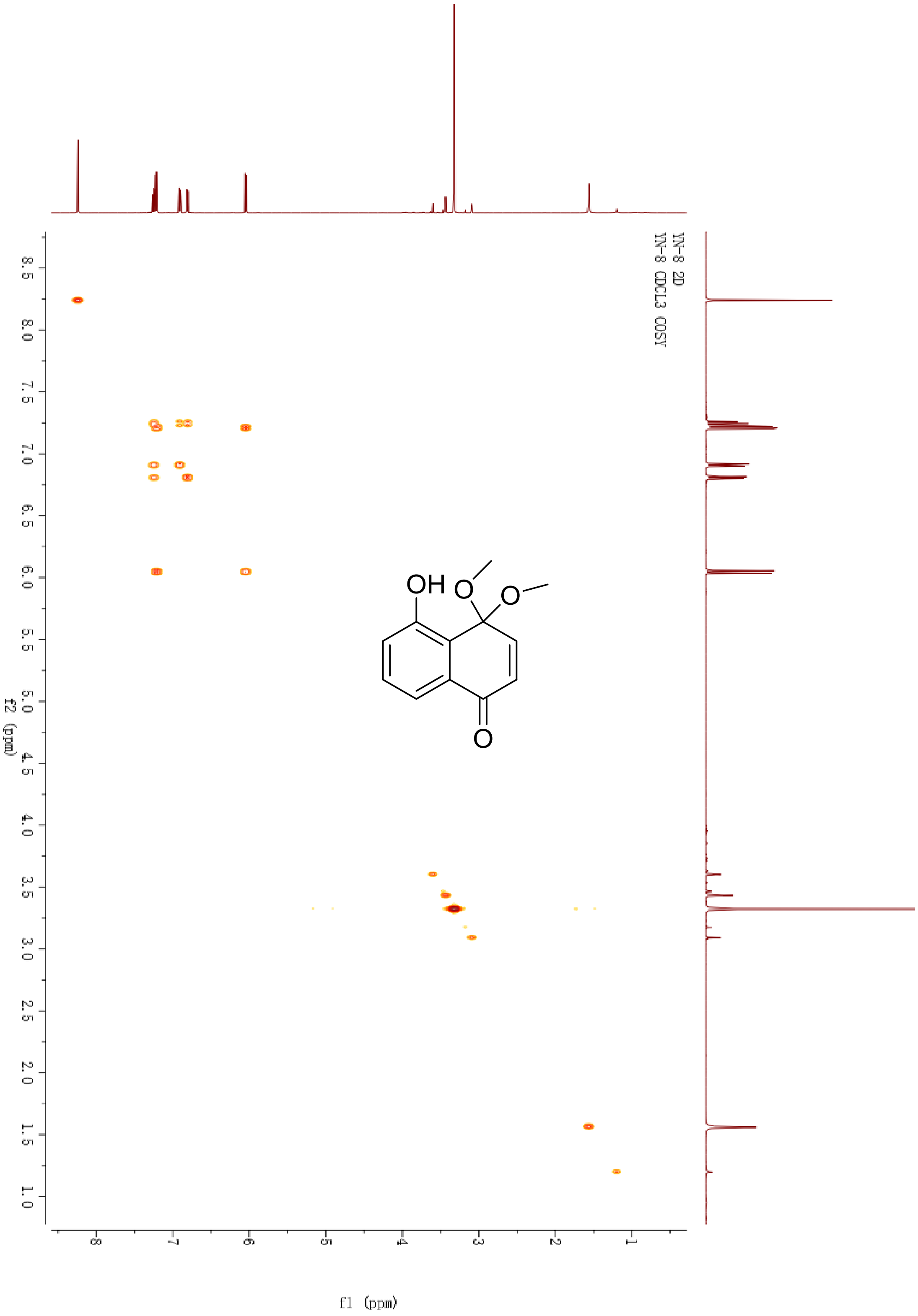

Figure S22. HRESIMS of naphthone C (3)

Formula Predictor Report - YN-8.lcd

Page 1 of 1

Data File: E:\DATA\2017\0413\YN-8.lcd

Elmt	Val.	Min	Max	Elmt	Val.	Min	Max	Elmt	Val.	Min	Max	Elmt	Val.	Min	Max	Use Adduct
H	1	0	150	O	2	0	50	S	2	0	0	Pt	2	0	0	Na
B	3	0	0	F	1	0	0	Cl	1	0	0					
C	4	0	100	Na	1	0	0	Br	1	0	0					
N	3	0	10	Si	4	0	0	I	3	0	0					

Error Margin (ppm): 10
HC Ratio: unlimited
Max Isotopes: all
MSn Iso RI (%): 75.00

DBE Range: -2.0 - 100.0
Apply N Rule: yes
Isotope RI (%): 1.00
MSn Logic Mode: AND

Electron Ions: both
Use MSn Info: yes
Isotope Res: 10000
Max Results: 10

Event#: 1 MS(E+) Ret. Time : 0.490 Scan# : 99

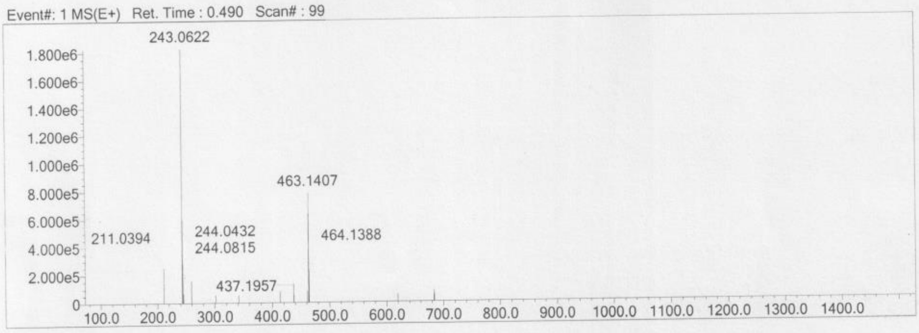

Measured region for 243.0622 m/z

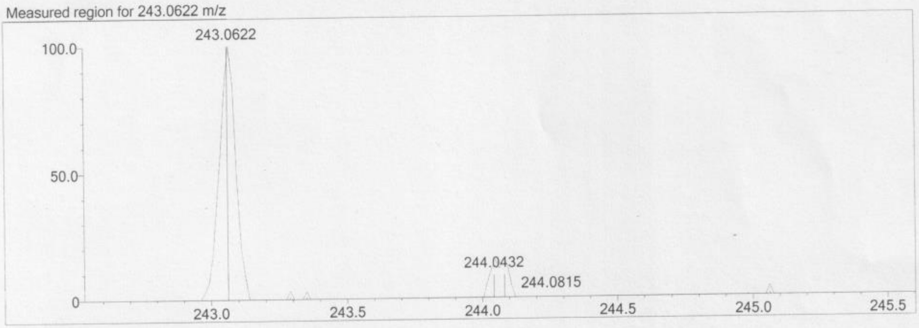

C12 H12 O4 [M+Na]+ : Predicted region for 243.0628 m/z

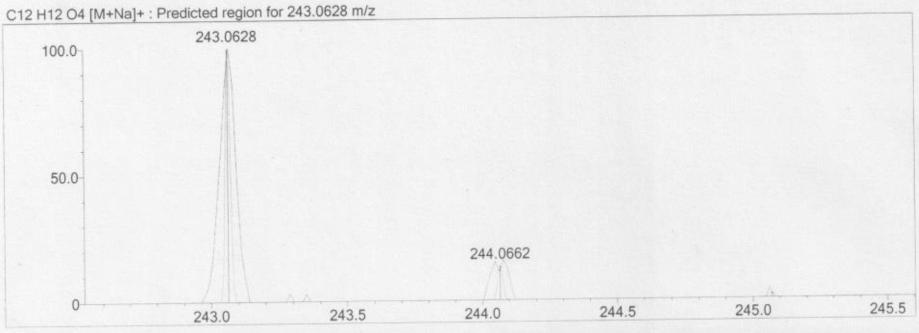

Formula (M)	Ion	Meas. m/z	Pred. m/z	Df. (mDa)	Df. (ppm)	DBE
C12 H12 O4	[M+Na]+	243.0622	243.0628	-0.6	-2.47	7.0

Figure S23. IR spectrum of naphthone C (3)

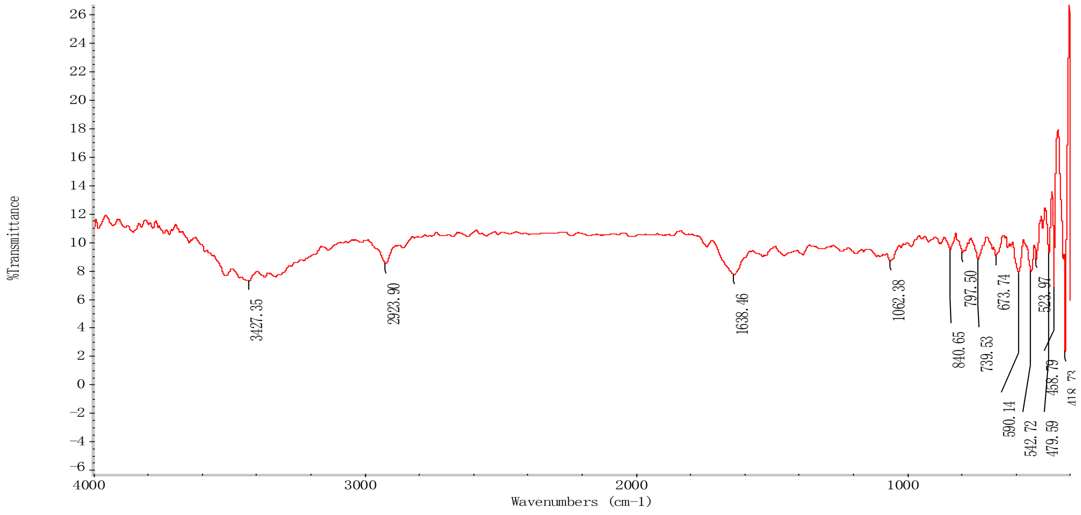

Supplement: Supplementary file 1 [file molecules-25-03898-s001.pdf]
